# Zeb1 mediates EMT/plasticity-associated ferroptosis sensitivity in cancer cells by regulating lipogenic enzyme expression and phospholipid composition

**DOI:** 10.1038/s41556-024-01464-1

**Published:** 2024-07-15

**Authors:** Annemarie Schwab, Zhigang Rao, Jie Zhang, André Gollowitzer, Katharina Siebenkäs, Nino Bindel, Elisabetta D’Avanzo, Ruthger van Roey, Yussuf Hajjaj, Ece Özel, Isabell Armstark, Leonhard Bereuter, Fengting Su, Julia Grander, Ehsan Bonyadi Rad, Arwin Groenewoud, Felix B. Engel, George W. Bell, Whitney S. Henry, José Pedro Friedmann Angeli, Marc P. Stemmler, Simone Brabletz, Andreas Koeberle, Thomas Brabletz

**Affiliations:** 1 Department of Experimental Medicine 1, Nikolaus-Fiebiger Center for Molecular Medicine, Friedrich-Alexander University of Erlangen-Nürnberg (FAU), Erlangen, Germany; 2 Michael Popp Institute and Center for Molecular Biosciences (CMBI), University of Innsbruck, Innsbruck, Austria; 3 Experimental Renal and Cardiovascular Research, Department of Nephropathology, Institute of Pathology, Friedrich-Alexander-Universität Erlangen-Nürnberg (FAU), Erlangen, Germany; 4 Comprehensive Cancer Center Erlangen-EMN (CCC ER-EMN), Bavarian Cancer Research Center (BZKF), Erlangen, Germany; 5 Whitehead Institute for Biomedical Research, Cambridge, MA USA; 6 Dept. of Biology, MIT, Cambridge, MA USA; 7 Rudolf Virchow Center for Integrative and Translational Bioimaging, University of Würzburg, Würzburg, Germany

**Keywords:** Cell death, Phospholipids, Epithelial-mesenchymal transition

## Abstract

Therapy resistance and metastasis, the most fatal steps in cancer, are often triggered by a (partial) activation of the epithelial–mesenchymal transition (EMT) programme. A mesenchymal phenotype predisposes to ferroptosis, a cell death pathway exerted by an iron and oxygen-radical-mediated peroxidation of phospholipids containing polyunsaturated fatty acids. We here show that various forms of EMT activation, including TGFβ stimulation and acquired therapy resistance, increase ferroptosis susceptibility in cancer cells, which depends on the EMT transcription factor Zeb1. We demonstrate that Zeb1 increases the ratio of phospholipids containing pro-ferroptotic polyunsaturated fatty acids over cyto-protective monounsaturated fatty acids by modulating the differential expression of the underlying crucial enzymes stearoyl-Co-A desaturase 1 (SCD), fatty acid synthase (FASN), fatty acid desaturase 2 (FADS2), elongation of very long-chain fatty acid 5 (ELOVL5) and long-chain acyl-CoA synthetase 4 (ACSL4). Pharmacological inhibition of selected lipogenic enzymes (SCD and FADS2) allows the manipulation of ferroptosis sensitivity preferentially in high-Zeb1-expressing cancer cells. Our data are of potential translational relevance and suggest a combination of ferroptosis activators and SCD inhibitors for the treatment of aggressive cancers expressing high Zeb1.

## Abstract (web summary)

Schwab, Rao et al. report that Zeb1 mediates enhanced ferroptosis sensitivity in cancer cells after EMT activation, associated with altered expression of selected lipogenic enzymes and an subsequent increase in the PUFA:MUFA ratio.

## Main

The two major obstacles in combating cancer progression are metastasis and therapy resistance, underscoring the urgent need for innovative therapeutic strategies. Both processes are often triggered by a transient and partial activation of the EMT programme in cancer cells, which is exerted by EMT transcription factors (EMT-TFs), mainly of the Zeb, Snail and bHLH families^1,2^. These highly plastic, partially mesenchymal cancer cells turned out to be the most crucial and fatal; they combine high tumorigenic and metastatic capacity with high resistance to any kind of current therapy modalities^3–5^, which makes them the ‘ultimate’ target in many cancer types. However, targeting these elusive cancer cell populations (either with a transient or intrinsic mesenchymal phenotype) has remained a considerable challenge until now.

A mesenchymal phenotype has been shown to predispose to a higher susceptibility to ferroptotic cell death^6^. Ferroptosis is an ancestral, highly conserved death pathway, depending on an iron and oxygen-radical-mediated peroxidation of phospholipids. Notably, such phospholipids must be composed of polyunsaturated fatty acids (PUFAs)^7–9^. In contrast to PUFAs, monounsaturated fatty acids (MUFAs) are resistant to peroxidation and excess MUFAs can even protect cells from ferroptosis and counteract PUFA biosynthesis^10,11^. Moreover, MUFAs can protect cells from death, as demonstrated for the MUFA-containing lipokine phosphatidylinositol (PI) (oleate (18:1/18:1))^12^. To prevent spontaneous ferroptosis, cells have efficient protection systems, leading to detoxification of lipid hydroperoxides (lipid reactive oxygen species (ROS)). Two well-known systems involve the enzymes GPX4 and FSP1, and their pharmacological inhibition can induce ferroptosis^7,13^. In contrast to apoptosis, the prevailing death pathway in differentiated cells (for example epithelial cells), ferroptosis can be executed predominantly in cells with a mesenchymal phenotype^6^, which is activated by the expression of EMT-TFs. Hereby, the expression of the EMT-TF Zeb1 has been associated with ferroptosis susceptibility, but the underlying molecular links are not known^6^. We and others have previously described that Zeb1 is a hallmark transcription factor of aggressive cancer types involved in all stages of fatal tumour progression, including therapy resistance and metastasis^1,4,14,15^. Therefore, the unexpected link of this metastasis-promoting EMT-TF with ferroptosis sensitivity offers a therapeutic window.

The aim of our study was to elucidate molecular mechanisms of the described Zeb1/EMT-associated ferroptosis sensitivity as basis for future therapeutic strategies.

## Results

### Ferroptosis sensitivity of mesenchymal cancer cells relies on Zeb1

**Fig. 1:**
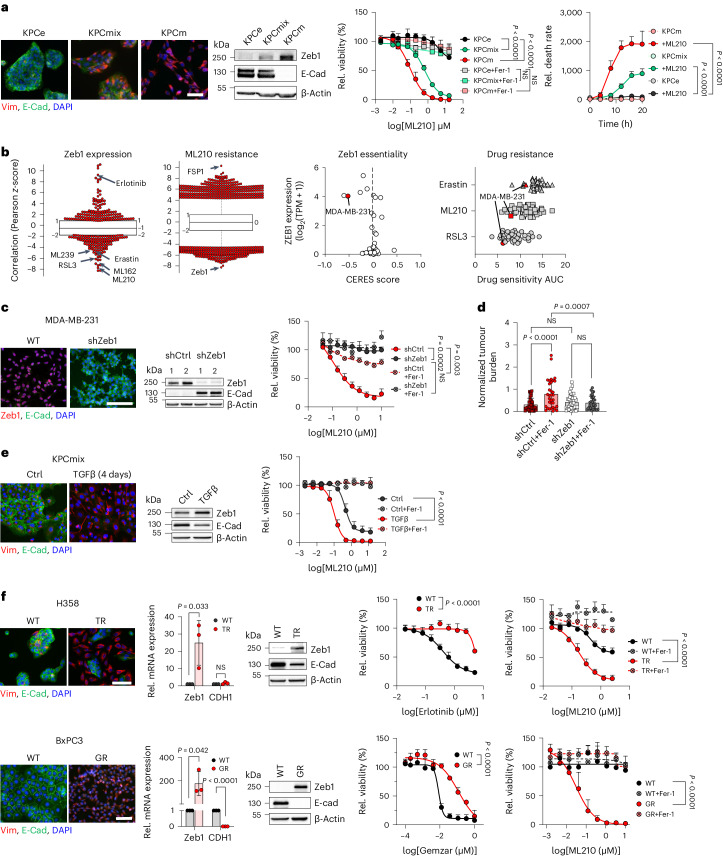
Zeb1 is important for increased ferroptosis sensitivity of mesenchymal cancer cells. **a**, Representative immunofluorescence and immunoblots of KPC cells, classified according to an epithelial (KPCe), mixed (KPCmix) or mesenchymal (KPCm) phenotype. Relative viability and death rate treated with ML210 (46 h) ± 1 µM ferrostatin-1 (Fer-1). Data are mean ± s.e.m. from *n* = 3 independent experiments, two-way analysis of variance (ANOVA) (with multiple comparisons). Scale bar, 50 µm. **b**, High expression of Zeb1 correlates with sensitivity to ferroptosis inducers, and inversely to the EGFR inhibitor erlotinib. ML210 sensitivity correlates with high Zeb1 and low FSP1 expression. Plotted values are *z*-scored Pearson’s correlation coefficients; line, median; box, 25th to 75th percentile; whiskers, 2.5th and 97.5th percentile expansion, available from the CTRP database (https://portals.broadinstitute.org/ctrp.v2.1/). CRISPR–Cas9 essentiality screens (CERES) identify *Zeb1* as an important gene in MDA-MB-231 cells (marked in red), which is among the most sensitive towards ferroptosis inducers. Plotted data were mined from Dependency Map (https://depmap.org/portal). **c**, Left, representative immunofluorescence and immunoblots of MDA-MB-231 shCtrl and shZeb1 cells. Right, relative viability after ML210 ± 1 µM Fer-1 treatment (72 h). Data are mean ± s.e.m. from *n* = 6 independent experiments (*n* = 4 for Fer-1 treatments), two-way ANOVA (with multiple comparisons). Scale bar, 50 µm. **d**, Relative tumour growth of MDA-MB-231 shCtrl and shZeb1 in zebrafish larvae, ±Fer-1 pretreatment. Data are mean ± s.e.m. from *n* = 39 shCtrl, *n* = 33 shCtrl + Fer-1, *n* = 36 shZeb1 ± Fer-1, ordinary one-way-ANOVA. **e**, Representative immunofluorescence and immunoblots in control or TGFβ-treated KPCmix cells. Relative viability after ML210 ± Fer-1 treatment (72 h). Data are mean ± s.e.m. from *n* = 3 independent experiments, two-way-ANOVA, scale bar, 50 µm. **f**, Representative immunofluorescence and immunoblots of H358 lung and BxPC3 pancreatic cancer cells, before (WT) and after resistance selection against the EGFR inhibitor erlotinib (Tarceva, TR) or the chemotherapeutic agent gemcitabine (Gemzar, GR). Relative cell viability measured 72 h after indicated treatments. Data are mean ± s.e.m. from *n* = 3 (H358) and *n* = 4 (BxPC3) independent experiments; two-way- ANOVA. Scale bar, 50 µm. Source data

**Extended Data Fig. 1:**
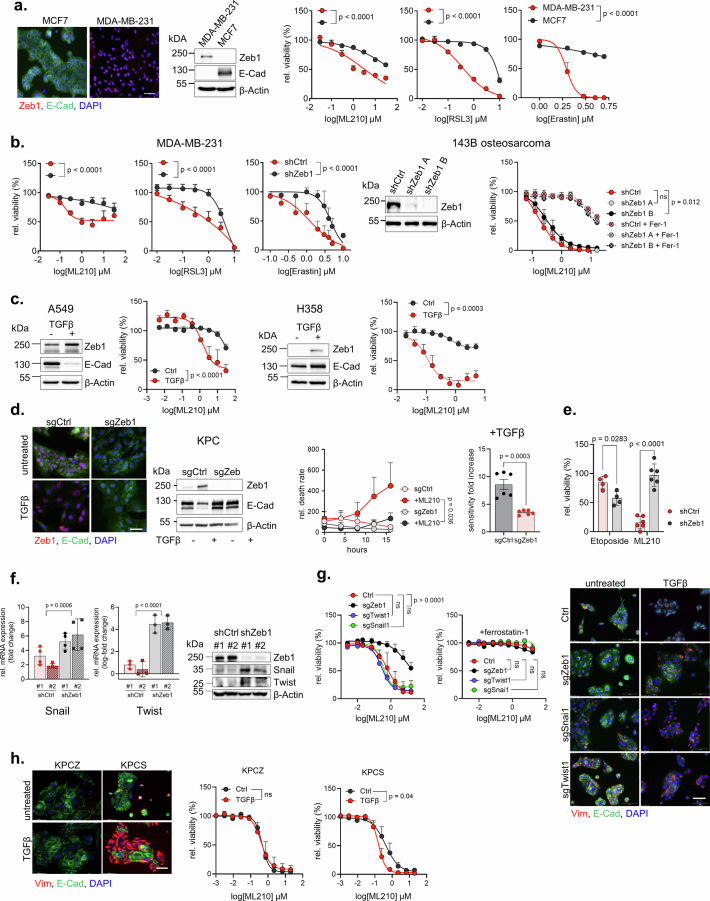
Zeb1 is important for increased ferroptosis sensitivity of mesenchymal cancer cells. **a**) Representative immunofluorescence and immunoblots for MCF7 and MDA-MB-231 breast cancer cells. Viability of MCF7 and MDA-MB-231 cells treated for 48 h with different ferroptosis inducers (FINs). n = 3 independent experiments, ordinary two-way ANOVA. **b**) Viability of MDA-MB-231 (shCtrl and shZeb1) cells treated for 48 h with indicated FINs. n = 3 (ML210), n = 5 (RSL3, shCtrl), n = 6 (RSL3, shZeb1; Erastin). Representative immunoblot in 143B osteosarcoma cells (shCtrl and shZeb1). Relative viability after ML210 treatment (72 h) ± Fer-1, from n = 6 independent experiments (n = 3 for Fer-1 treatments), ordinary two-way ANOVA. **c**) Representative immunoblots and ML210 viability assays (72 h) in control- or TGFβ-treated A549 and H358 cells, for 4 and 9 days respectively. n = 3 independent experiments, ordinary two-way ANOVA. **d**) Representative immunofluorescence and immunoblots in control or Zeb1ko KPCe cells. Cell death for syngeneic KPCe clones (sgCtrl and sgZeb1) after ML210 treatment (3 µM), n = 3 independent experiments, ordinary two-way ANOVA. TGFβ-induced ferroptosis sensitization (relative change of IC50 for ML210) in control or Zeb1ko KPCmix cells. n = 3 independent experiments, two-tailed unpaired student t-test (bar graph). **e**) Viability assay of shCtrl or shZeb1 MDA-MB-231 cells after treatment with either the chemotherapeutic agent etoposide (10 µM, n = 4) or ML210 (5 µM, n = 6) for 48 h, independent experiments, ordinary two-way ANOVA. **f**) mRNA and protein expression of EMT-TFs in the indicated MDA-MB-231 clones. n = 4 for Snail, n = 3 for Twist, ordinary one-way ANOVA. **g**) Representative immunofluorescence in control KPCe cells or after CRISPR-mediated knockout of indicated EMT-TFs treated with TGFβ (5 ng/ml, 5 days). Relative viability measured after 72 h ML210 treatment ± Fer-1. n = 3 independent experiments, ordinary two-way ANOVA. **h**) Representative immunofluorescence cells derived from tumours with a conditional knockout for Zeb1 (KPCZ) or Snail (KPCS) ± TGFβ (5 ng/ml, 5 days). Relative viability 72 h after ML210 treatment (72 h). n = 3 independent experiments, ordinary two-way ANOVA. All data are presented as mean ± SEM, scale bar of all presented fluorescence images is 50 µM.

We investigated whether different ways of EMT activation all lead to increased ferroptosis susceptibility and if this relies on common key factors. First, we analysed cancer cell models with intrinsically different phenotypes. The KPC genetic mouse model gives rise to metastatic pancreatic cancers with highly variable phenotypes^15,16^. Ferroptosis sensitivity in various cell lines isolated from such tumours was closely linked to their intrinsic phenotype, with mesenchymal tumour cells showing the highest susceptibility (Fig. 1a). Moreover, GPX4 inhibition selects for an epithelial phenotype in KPCmix cell lines with a mixed, plastic phenotype (Supplementary Video 1). The same association is evident in established human cancer cell lines, for example from breast cancer, as exemplified for the lines MDA-MB-231 (mesenchymal) and MCF7 (epithelial) (Extended Data Fig. 1a). Analyses of published datasets confirmed a strong association of sensitivity to various ferroptosis-inducing compounds with high expression of the EMT-TF Zeb1 (Fig. 1b). CRISPR–Cas9 essentiality screens (CERES) identify Zeb1 as an important gene in MDA-MB-231 cells, one of the most ferroptosis sensitive cancer cell lines (Fig. 1b). Accordingly, depletion of Zeb1 reduced ferroptosis susceptibility (Fig. 1c and Extended Data Fig. 1b). Zeb1 dependency could be confirmed in vivo in a zebrafish model. Here tumour growth was strongly enhanced by ferrostatin-1 for MDA-MB-231 wild-type cells, indicating spontaneous ferroptosis, but not for Zeb1-depleted cells (shZeb1) (Fig. 1d).

In human cancers a partial mesenchymal state is often transiently activated in plastic epithelial cancer cells by environmental factors, for example TGFβ, or in the course of therapy resistance. This clinically relevant type of a transient EMT activation by TGFβ also increased the sensitivity to ferroptosis in various epithelial cancer cell types (Fig. 1e and Extended Data Fig. 1c), which could again be attenuated by depletion of Zeb1 (Extended Data Fig. 1d). The same association of an acquired mesenchymal state with ferroptosis sensitivity was detected in various EMT/Zeb1-associated therapy resistance models established in our laboratory^17^. Epithelial-type H358 lung cancer cells are highly sensitive to inhibition of EGFR-signalling, but resistant to ferroptosis. Selection of cells resistant to the EGFR inhibitor erlotinib (Tarceva) strongly enriches for a Zeb1^high^ mesenchymal phenotype, which acquired a high ferroptosis sensitivity (Fig. 1f). Notably, this is consistent with the reciprocal sensitivity to ferroptosis-inducing compounds and erlotinib depending on the expression of Zeb1 (Fig. 1b). The same behaviour was observed for the pancreatic cancer cell line BxPC3 and sublines made resistant to the chemotherapeutic agent gemcitabine (Fig. 1f), as well as for MDA-MB-231 cells, where Zeb1 expression determines sensitivity to ferroptosis, but Zeb1-depleted cells, while highly resistant to ferroptosis, increase sensitivity to the chemotherapeutic agent etoposide (Extended Data Fig. 1e). These results are in line with data described for drug tolerant persister cells, which also show a high sensitivity to ferroptosis^18^. Of note, Zeb1 depletion in MDA-MB-231 led to an increase in the expression of the EMT-TFs Snail and Twist1 (Extended Data Fig. 1f), indicating that Snail and Twist1 cannot substitute Zeb1 for maintaining a high ferroptosis susceptibility. This finding was confirmed in the pancreatic cancer cell models, where only knockout of Zeb1, but not of Snail and Twist1 decreased ferroptosis sensitivity in TGFβ-treated KPCe cells (Extended Data Fig. 1g) and for cell lines generated from autochthonous tumours grown in mice with conditional knockout of Zeb1 versus Snail, as previously described^15^ (Extended Data Fig. 1h). In both model systems, the effects were coupled to a block of EMT induction only in Zeb1-depleted cells, supporting the strong link of a mesenchymal phenotype with ferroptosis susceptibility.

In summary, different ways of EMT activation in cancer cells increase ferroptosis sensitivity for which the EMT-TF Zeb1 is a crucial underlying factor.

### Zeb1 enriches PUFAs in phospholipids and enhances peroxidation

**Fig. 2:**
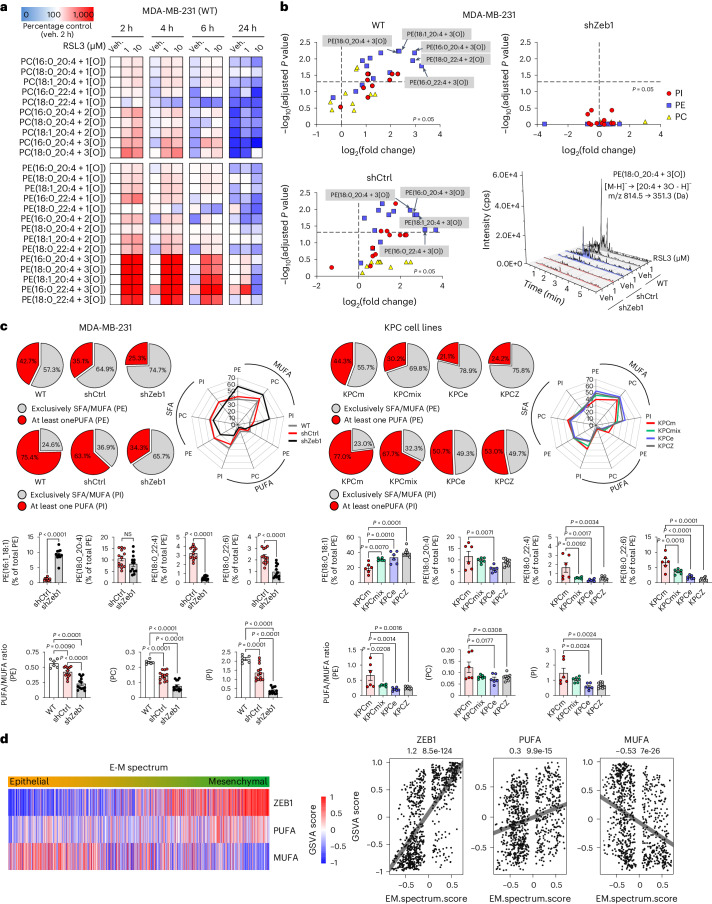
Zeb1 sensitizes to phospholipid peroxidation and upregulates PUFAs. Profiling of phospholipids and oxidized phospholipids in MDA-MB-231 cells after the indicated treatment and in various KPC cell lines. **a**, Time-dependent induction of the formation of oxidized phosphatidylcholine (PC) and phosphatidylethanolamine (PE) species by the GPX4 inhibitor RSL3 in MDA-MB-231 cells (wild type, WT). The colour scheme represents the mean percentage change against vehicle (Veh) from *n* = 3 independent experiments. **b**, Volcano plots show the changes in the amount of RSL3-induced oxidized phosphatidylinositol (PI), PE and PC species. Fold changes were calculated from the absolute intensities of species derived from cells treated with RSL3 (1 µM, 2 h) against Veh. *n* = 3 independent experiments, multiple two-tailed unpaired *t*-test with 5% false discovery rate. Exemplary extracted UPLC–MS/MS chromatogram for oxidized PE(18:0_20:4 + 3[O]) species from WT, shCtrl and shZeb1 upon 2 h treatment with Veh or RSL3 (1 µM). cps, counts per second. **c**, Effect of Zeb1 depletion (shZeb1) on the fatty acid distribution of analysed phospholipid species in MDA-MB-231 cells (left) and in KPC cell lines with different phenotypes (right). Pie charts indicate the relative abundance (% of total PE) of SFA/MUFA- and PUFA-containing PE or PI species (SFA). Bar charts show the relative abundance of exemplary PE species as well as the PUFA:MUFA ratio in phospholipids. Radar plots show the proportion of PUFA versus MUFA and SFA in PE, PC or PI. Data are given as mean (pie charts and radar plots) or mean ± s.e.m. (bar charts) from *n* = 6–13 (*n* = 6 for WT; *n* = 13 for shCtrl and shZeb1; *n* = 6 for KPCm, KPCmix and KPCe; *n* = 9 for KPCz) independent experiments; two-tailed unpaired Student’s *t*-test or ordinary one-way ANOVA. **d**, Heat map and scatter-plots showing the expression after a gene set variation analysis (GSVA) of Zeb1 as well as PUFA and MUFA-related gene signatures along the epithelial to mesenchymal (E–M) spectrum of 500 human metastatic cancers. Source data

**Extended Data Fig. 2:**
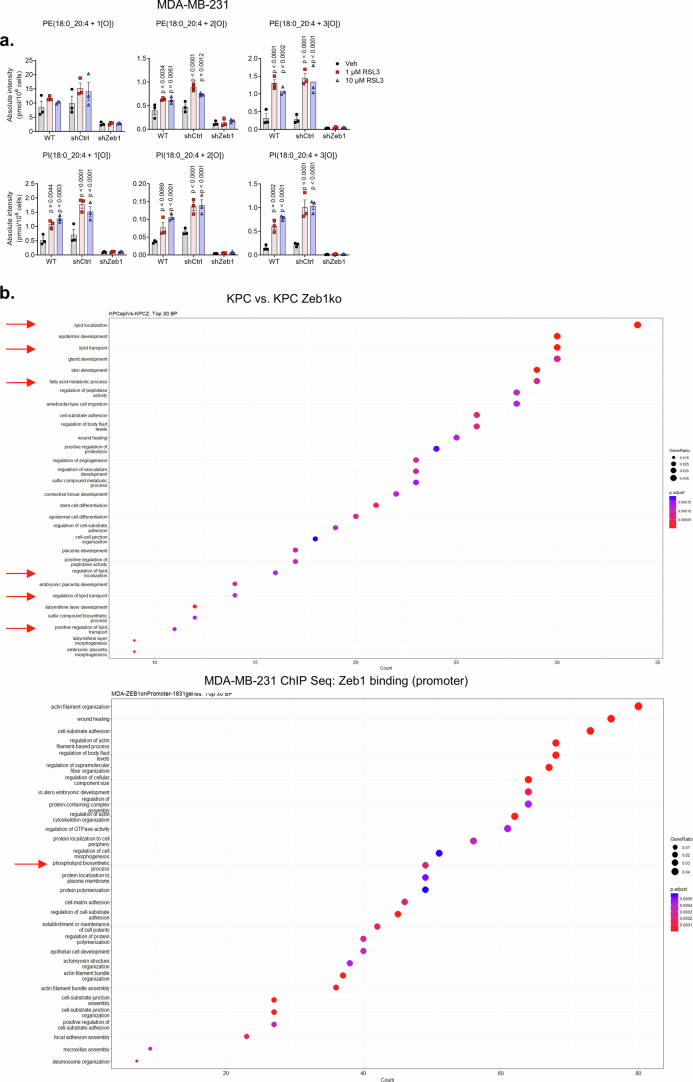
Zeb1 and enhances phospholipid peroxidation and modulates lipid metabolism. **a**) Zeb1 is critical for RSL3-induced phospholipid peroxidation in MDA-MB-231 cells. Exemplary oxidized PE or PI(18:0_20:4) (PE, phosphatidylethanolamines; PI: phosphatidylinositol) species in MDA-MB-231 cells (WT, shCtrl, shZeb1) treated with RSL3 at the indicated concentrations for 2 h. n = 3 independent experiments, ordinary two-way ANOVA. **b**) Zeb1 induces massive metabolic reprogramming. GO term analysis (top 30 list) representing transcriptional changes (upper panel) and Zeb1 promoter binding (determined by Zeb1 ChIP-seq, lower panel) in control vs. Zeb1ko KPCe cells. Arrows mark GO terms related to fatty acid metabolism.

We further investigated the underlying mechanisms of an EMT/Zeb1-coupled ferroptosis sensitivity. In this study we focused on the proportion and (per)oxidation of fatty acid species, as ferroptosis depends on the peroxidation of membrane phospholipids containing PUFAs^7,8^^,^^10^^,^^19^. We demonstrated that GPX4 inhibition in MDA-MB231 cells (Zeb1^high^) led to a rapid (per)oxidation of PUFA-containing phospholipids (Fig. 2a). Most responsive were phosphatidylethanolamine (PE) species, which contain (per)oxidized arachidonic acid or adrenic acid (AdA) that have three oxygens incorporated (3[O]), all known to be critical targets for executing ferroptosis^19^. By contrast, cells depleted of Zeb1 showed an almost complete absence of oxidized phospholipids, in particular of the species relevant for ferroptosis (Fig. 2b and Extended Data Fig. 2a).

**Extended Data Fig. 3:**
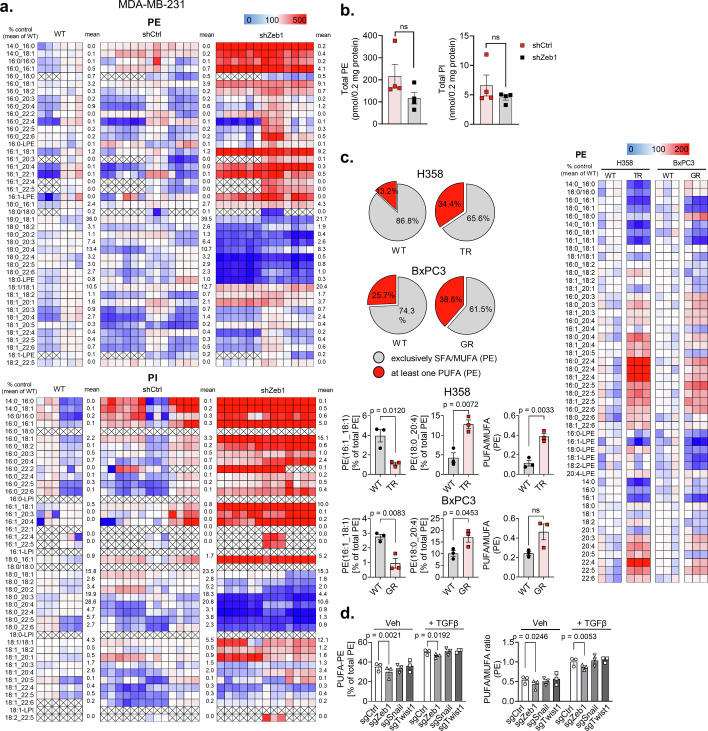
Zeb1 upregulates phospholipid PUFA ratios. **a**) Zeb1 shapes the phospholipid profile in MDA-MB-231 cells. Relative abundance of individual PE or PI phospholipids (% of total PE or PI) in MDA-MB-231 (WT, shCtrl or shZeb1) cells. The colour scheme shows the percentage change of relative intensities in shCtrl and shZeb1 compared to WT. Numbers indicate the mean proportions relative to the sum of all PE or PI species analysed (100%). Individual data for n = 6 (WT) or n = 13 (shCtrl, shZeb1) independent experiments are shown. **b**) Total PE and PI content in MDA-MB-231 (shCtrl and shZeb1) cells. Data are presented as mean ± SEM from n = 4 individual experiments, two-tailed unpaired student t-test. **c**) Fatty acid distribution of PE species in H358 lung cancer cells and BxPC3 pancreatic cancer cells (WT) cells selected for resistance against the EGFR inhibitor erlotinib/Tarceva (TR) or the chemotherapeutic gemcitabine (GR). Pie charts indicate the relative abundance (% of total PE) of SFA and/or MUFA- vs. PUFA-containing PE species. Bar charts show the relative abundance of exemplary PE species as well as the PUFA/MUFA ratio in PE. The heat maps show the percentage change in the relative abundances of either PE species or the proportion of individual fatty acids in PE. Data are given as mean (pie charts), mean ± SEM (bar charts) or individual values (heat maps), from n = 3 independent experiments, two-tailed unpaired student t-test. **d**) Bar charts showing the relative abundance of PUFA-containing PE species and the PUFA/MUFA ratio PE species in the indicated KPCe CRISPR-knockout cells with or without TGFβ treatment for 5 days and are given as mean ± SEM from n = 3 independent experiments, repeated measures, two-way ANOVA.

Zeb1 has previously been implicated in the control of adipocyte differentiation^20^ and lipid metabolism^21^. Analyses of OMICs datasets revealed that Zeb1 expression controls a massive metabolic reprogramming, including the regulation of fatty acid biosynthesis and metabolism (Extended Data Fig. 2b). As described above, differential fatty acid biosynthesis is of high relevance, as different long-chain PUFAs are oxidized during ferroptosis^19,22–24^ and thus the PUFA:MUFA ratio in phospholipids is critical for ferroptosis sensitivity. Notably, phospholipid species containing PUFAs, including the ferroptosis-relevant PE(18:0_20:4) and PE(18:0_22:4), as well as the PUFA:MUFA ratio were highly enriched in Zeb1^high^ mesenchymal-type cancer cells (MDA-MB-231 and KPCm), but reduced upon Zeb1 depletion, as well as in epithelial (KPCe) cancer cells (Fig. 2c and Extended Data Fig. 3a). By contrast, the proportion of MUFAs in phospholipids was increased. Thereby Zeb1 depletion results in a reduction of phospholipids with longer-chain fatty acids (18:0) and an accumulation of those with shorter-chain fatty acids, particularly myristate (14:0) and palmitate (16:0) (Extended Data Fig. 3a). By contrast, the total phospholipid content was only weakly affected (Extended Data Fig. 3b). Notably, the proportion of major MUFA species, for example 16:1 and 18:1, including the stress-protective lipokine PI(18:1/18:1)^12^, was substantially upregulated upon Zeb1 depletion. These data indicate that Zeb1 depletion leads to a reduced incorporation of PUFAs into phospholipids and/or a reduction in both fatty acid chain elongation and fatty acid desaturation. The same Zeb1-associated modulation of PUFA-containing phospholipids was detected in drug-resistant H358 and BxPC3 cells (Extended Data Fig. 3c), which acquired a mesenchymal, Zeb1^high^ phenotype and high ferroptosis sensitivity (Fig. 1e). Again, the PUFA:MUFA ratio was only significantly affected by a depletion of Zeb1, but not of Snail and Twist1 (Extended Data Fig. 3d). These experimental data are supported by analyses of human cancer datasets^25^, which we stratified along the epithelial–mesenchymal axis, showing that mesenchymal-type cancers have higher expression levels of PUFA-related gene signatures and lower levels of MUFA-related gene signatures (Fig. 2d).

In summary, Zeb1 expression has a strong impact on phospholipid fatty acid (per)oxidation and composition, particularly by raising the membrane PUFA:MUFA ratio.

### Zeb1 regulates the expression of enzymes controlling the PUFA:MUFA ratio

**Fig. 3:**
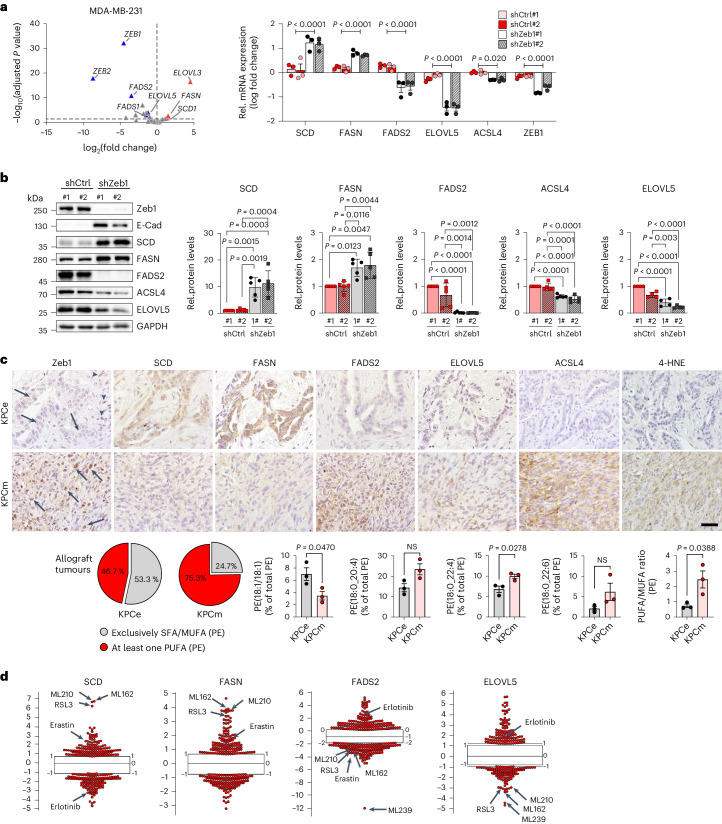
Zeb1 regulates the expression of enzymes crucial for adjusting the PUFA:MUFA ratio in phospholipids. **a**, Volcano plot showing mRNA expression changes of indicated factors in MDA-MB-231 (shZeb1 versus shCtrl) cells deduced from transcriptome analysis (left). Fold changes were calculated as shZeb1 versus shCtrl, adjusted *P* values were determined by multiple unpaired *t*-tests with 5% false discovery rate. Changes in mRNA expression are plotted as mean ± s.e.m. from *n* = 3 independent experiments; multiple unpaired *t*-test after grouping of independent clones. **b**, Immunoblots and quantification of lipogenic enzymes in MDA-MB-231 shCtrl and shZeb1 cells. Proteins were normalized to their respective GAPDH from the same blot, followed by normalization to MDA-MB-231 shCtrl clone no. 1, Bar charts are plotted as mean ± s.e.m. from *n* = 5 (except ELOVL5 *n* = 4), ordinary one-way ANOVA. **c**, Representative immunohistochemistry images showing the expression of Zeb1, the indicated lipogenic enzymes, 4-HNE as marker for lipid oxidation as well as fatty acid distribution of analysed phospholipid species in grafted KPC tumours with indicated phenotypes (KPCe, differentiated; KPCm, undifferentiated/mesenchymal) from *n* = 3 tumours each. Arrows indicate tumour cells, arrowheads indicate fibroblasts, specific staining is shown in brown. Scale bar, 100 µm. PE profile in KPC tumour allografts. Pie charts indicate the relative abundance (% of total PE) of SFA/MUFA- and PUFA-containing PE species. Bar charts show the relative abundance of exemplary PE species as well as the PUFA:MUFA ratio in PE. Data are given as mean or mean ± s.e.m. from *n* = 3 tumours; two-tailed unpaired Student’s *t*-test. **d**, High expression of the indicated lipogenic enzymes in cancer cell lines shows reciprocal correlation between sensitivity to EGFR inhibitor erlotinib and various ferroptosis-inducing compounds. Plotted values are *z* scored Pearson’s correlation coefficients between enzyme expression and drug resistance as the area under the curve of 481 compounds in 860 cancer cell lines, available from the CTRP database (https://portals.broadinstitute.org/ctrp.v2.1/). Line, median; box, 25th to 75th percentile; whiskers, 2.5th and 97.5th percentile expansion.

**Fig. 4:**
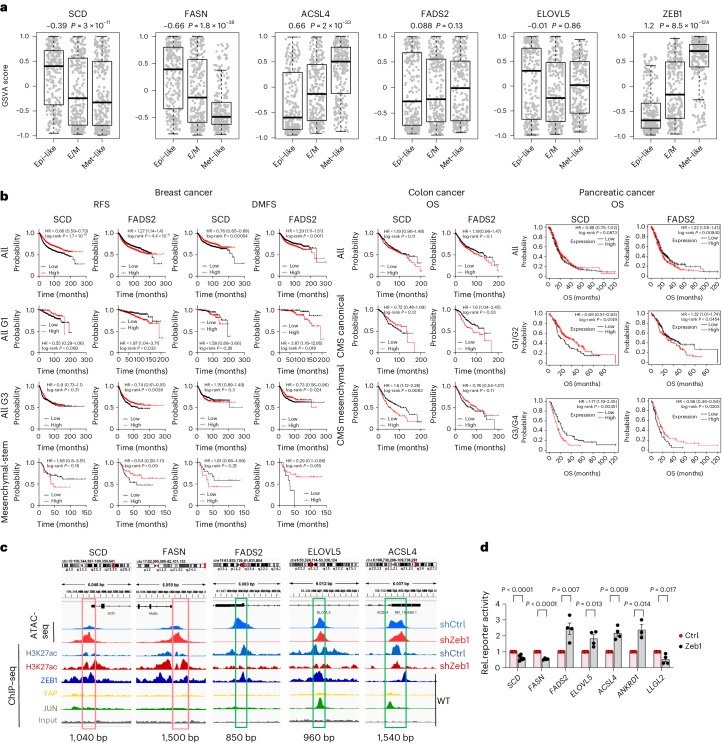
Zeb1 regulates the transcription of enzymes crucial for adjusting the PUFA/MUFA ratio in phospholipids. **a**, Box plots showing the expression of indicated factors after GSVA along the E–M spectrum of 500 human metastatic cancers. **b**, Kaplan–Meier plots (log-rank test) from survival analyses of human breast, colon and pancreatic cancers showing relapse-free survival (RFS), distant-metastasis-free survival (DMFS) and overall survival (OS) based on the expression of SCD or FADS2. Patients were not selected (all) or selected according to histological tumour grade or molecular subgroup. *n* = numbers with high (black) or low (red) expression (HR, hazard ratio). **c**, Peaks at the genomic loci of the indicated lipogenic enzymes derived from ATAC-seq and ChIP–seq for the active histone mark H3K27ac (comparing MDA-MB-231 shCtrl and shZeb1), as well as ChIP–seq for Zeb1, YAP and JUN (only in MDA-MB-231 WT). Note that Zeb1 binding peaks are detected in all loci, but ATAC-seq peaks and H3K27ac-seq peaks, indicating open chromatin and transcriptionally active regions, behave differently for repressed genes (SCD and FASN, red box) and activated genes (FADS2, ELOVL5 and ACSL4, green box). Boxes and base pair (bp) numbers indicate sizes and regions cloned in luciferase reporter constructs used in **b**. **d**, Luciferase reporter assays for the indicated regulatory elements of the *SCD*, *FASN*, *FADS2*, *ELOVL5* and *ACSL4* genes. Zeb1 expression was manipulated by overexpression in MCF7 (low endogenous expression). Reporter constructs of known Zeb1 target genes (*ANKRD1* activated and *LLGL2* repressed by Zeb1) were used as controls. Data are calculated as relative ratio to empty vector (Ctrl) and given as mean ± s.e.m. from *n* = 4 independent experiments (except for *SCD n* = 6 and *LLGL2 n* = 3), two-tailed unpaired Student’s *t-*test.

**Extended Data Fig. 4:**
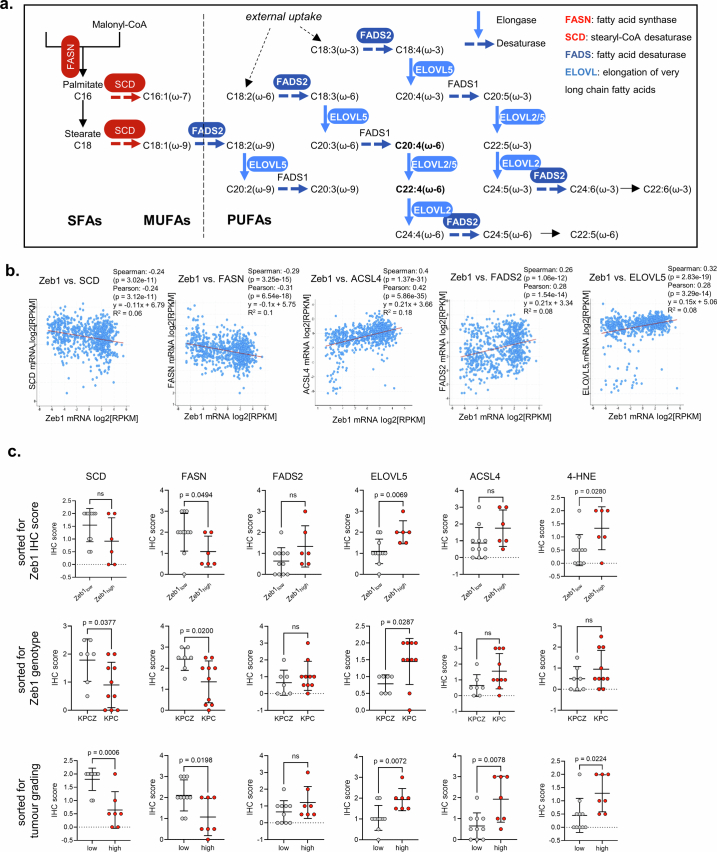
Zeb1 regulates the expression of lipogenic enzymes crucial for adjusting the PUFA/MUFA ratio. **a**) Simplified scheme of fatty acid biosynthesis pathways including relevant lipogenic enzymes. Note that most PUFAs, including those considered most relevant for the execution of ferroptosis (in bold) are not generated de novo, but are derived from essential fatty acids (C18:3 (ω-3) / linolenic acid and C18:2 (ω-6) / linoleic acid) taken up by the cell. **b**) Correlation of lipogenic enzymes with Zeb1 expression in 732 solid cancer cell lines from the CCLE database using cBioPortal (http://www.cbioportal.org/; https://sites.broadinstitute.org/ccle/). Shown are Spearman and Pearson correlations, derived from two-tailed t-test. **c**) IHC scores of indicated factors in allograft mouse tumours. Tumours were grouped in three different ways: 1. Zeb1-IHC Score (Zeb1low ≤ 1 vs Zeb1high > 1), 2. Zeb1 genotype (KPC, Zeb1 wt vs KPCZ, Zeb1ko), 3. histological tumour grade (low ≤ 2 vs high > 2). Bar graphs are presented as mean ± SD from n = 17 individual tumours, two-tailed unpaired student t-test.

The synthesis of saturated fatty acids (SFAs), MUFAs and PUFAs takes place in highly interdependent pathways involving key lipogenic enzymes that perform stepwise elongation and desaturation of fatty acids^26,27^ (Extended Data Fig. 4a). Notably, PUFAs critical for the execution of ferroptosis (for example, C20:4,ω-6/arachidonic acid and C22:4,ω-6/AdA) cannot be generated de novo, but require the uptake of essential fatty acids (linoleic acid or linolenic acid). For incorporation into phospholipids, fatty acids must be coupled to coenzyme A (CoA) by long-chain acyl-CoA synthetase (ACSL) family members^23^. We subsequently analysed the Zeb1-regulated transcriptome in MDA-MB-231 cells^28^ for underlying key enzymes. Elongation of ELOVL5, the crucial enzyme for medium-to-long-chain PUFA synthesis^29^, FADS2, critical for further desaturation of PUFAs^27^, as well as ACSL4, which initiates PUFA incorporation into phospholipids^30^, were downregulated in Zeb1-depleted cells (Fig. 3a,b). By contrast, SCD and FASN, which are critical for MUFA synthesis^31,32^, were upregulated upon Zeb1 depletion. This is also reflected by the correlation (ELOVL5, FADS2 and ACSL4) and anti-correlation (FASN and SCD) of these enzymes with Zeb1 expression in cancer cell lines (Extended Data Fig. 4b). A similar expression pattern was found in mouse tumour allografts, where low-grade differentiated tumours (KPCe and KPCZ, Zeb1^low^) expressed higher levels of SCD and FASN, and lower levels of FADS2, ELOVL5 and ACSL4, whereas cancer cells in high-grade undifferentiated tumours (KPCm and Zeb1^high^) showed a reverse expression pattern (Fig. 3c and Extended Data Fig. 4c). Moreover, the differential expression of these lipogenic enzymes correlated with the PUFA:MUFA ratio (Fig. 3c). Notably, as shown for Zeb1 (Fig. 1b), the expression level of ELOVL5 and FADS2 predicts high sensitivity to ferroptosis-inducing drugs and resistance to targeted drugs such as erlotinib, whereas expression of SCD and FASN correlated with inverse behaviour (a dataset for ACSL4 was not publicly available) (Fig. 3d). These experimental data are supported by analyses of human cancer datasets^25^, showing that mesenchymal-type cancers, exhibit lower *SCD* and *FASN* mRNA expression, but particularly higher expression of *ACSL4* (Fig. 4a). Furthermore, by applying the KM-Plotter analysis platform^33^, we found a correlation of SCD with better and of FADS2 expression with worse survival of individuals with breast cancer (Fig. 4b). A similar pattern was seen for the fraction of only low-grade (G1) tumours. Of note, an inverse correlation was detected for high-grade (G3) and particularly for the rare subtype of mesenchymal (stem)-like tumours. An explanation for this might be given by our data. If undifferentiated (mesenchymal)-type tumours express higher levels of a PUFA synthesis enzyme, they will have a higher ferroptosis susceptibility, which might be particularly relevant during dissemination in the bloodstream and thus would lead to lower metastasis, the major cause of death in breast cancer. In differentiated G1 (Zeb1^low^) tumours this would not be relevant due to their general low ferroptosis sensitivity. Indeed, the same correlation pattern could be detected for distant-metastasis-free survival, supporting this hypothesis. Finally, we detected a similar switch from poor to better survival correlated with FADS2 expression in high-grade pancreatic and mesenchymal-type colorectal cancers (Fig. 4b).

**Extended Data Fig. 5:**
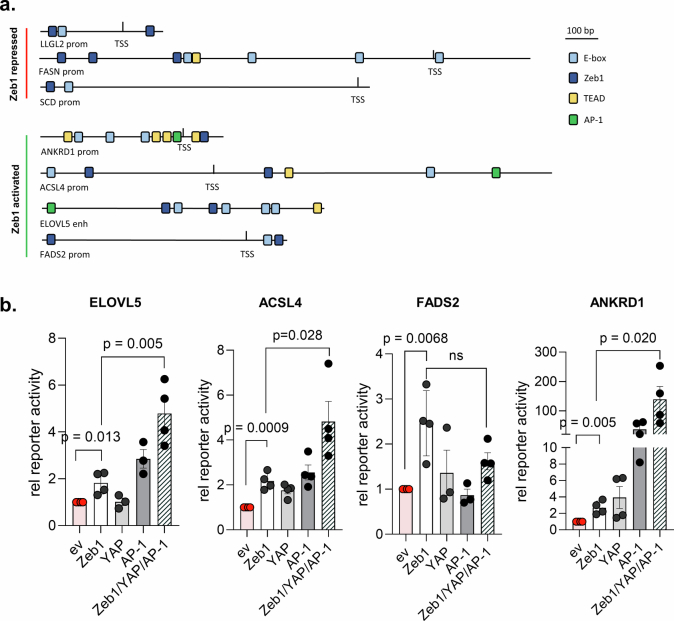
Zeb1 regulates the transcription of enzymes crucial for adjusting the PUFA/MUFA ratio in phospholipids. **a**) Schemes of genomic regions with predicted binding sites for indicated factors of Zeb1 repressed and activated genes (TSS = transcriptional start site). **b**) Luciferase reporter assays for the indicated regulatory elements of Zeb1 activated genes (ELOVL5, FADS2, and ACSL4) after co-transfection of the indicated transcription factors in MCF7 (low endogenous expression). A reporter constructs of the known Zeb1 activated target gene ANKRD1 was used as positive control. Data are given as relative ratio to empty vector (ev) and are presented as mean ± SEM from n = 3–6 independent experiments, two-tailed unpaired student t-test.

We next investigated a direct transcriptional gene regulation of those lipogenic enzymes by Zeb1, which is able to both activate and repress genes, depending on the genomic context^28,34^. Analyses of chromatin immunoprecipitation with high-throughput sequencing (ChIP–seq) data for Zeb1 and the histone mark H3K27ac (indicating high transcriptional activity), as well as assay for transposase-accessible chromatin using sequencing (ATAC-seq) data (indicating open chromatin) generated in MDA-MB-231 cells^28^ revealed that Zeb1 binds to and regulates all five respective gene loci (Fig. 4c). Zeb1-binding regions were associated with an active, open (*FADS2*) or inactive, closed (*SCD* and *FASN*) chromatin state in Zeb1^high^-expressing cancer cells, and an inverse pattern in Zeb1-depleted cells. *ELOVL5* and *ACSL4* regulatory regions showed an open chromatin independent of Zeb1 expression. Binding of the active mark H3K27ac correlated with the mRNA expression patterns, showing high binding for *FADS2, ELOVL5* and *ASCL4* in Zeb1^high^ cells (shCtrl) and high binding for *SCD* and *FASN* in Zeb1-depleted cells. Luciferase reporter assays for the respective regulatory regions revealed a direct activation by Zeb1 through both *ACSL4*, *FADS2* and *ELOVL5* regulatory regions. By contrast, the activity of *SCD* and *FASN* gene regions was decreased by Zeb1 overexpression (Fig. 4d). We previously described that Zeb1 cooperates with the transcription factors YAP/TEAD and AP-1 to activate transcription of certain gene panels^28,34^. We detected potential binding regions and respective ChIP–seq peaks for all three transcription factors in the regulatory regions of the *ELOVL5* and *ACSL4* genes, but not of *FADS2* (Fig. 4c and Extended Data Fig. 5a). A cooperative reporter gene activation by Zeb1, YAP and AP-1 was confirmed for *ELOVL5* and *ACSL4*, but as predicted, not seen for *FADS2* (Extended Data Fig. 5b). These data indicate a direct transcriptional regulation of *ACSL4*, *FADS2* and *ELOVL5* expression (activation) as well as *SCD* and *FASN* expression (repression) by Zeb1.

### PUFA:MUFA ratio-adjusting enzymes control ferroptosis sensitivity

Although the availability of PUFA-containing phospholipids is only one factor in the complex regulation of ferroptosis^7,8^, we here focused on the question, whether manipulation of Zeb1-regulated key enzymes for PUFA and MUFA synthesis can alter the ferroptosis sensitivity, as suggested by their reciprocal association with sensitivity to ferroptosis-inducing compounds (Fig. 3d). We focused on SCD and FADS2, for which selective pharmacological inhibitors are commercially available. Particularly blocking SCD activity, is of potential translational interest to promote ferroptosis susceptibility for cancer therapy.

**Fig. 5:**
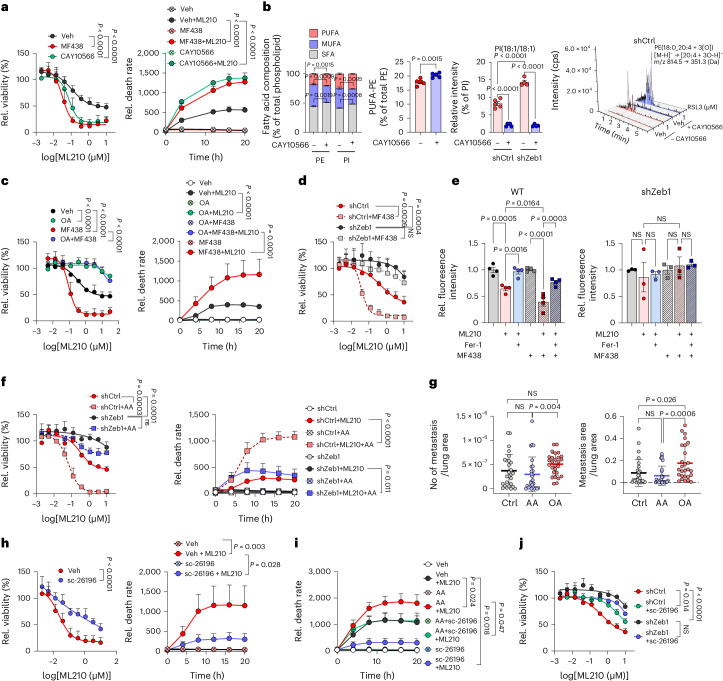
Inhibition of crucial enzymes adjusting the PUFA:MUFA ratio affects ferroptosis sensitivity in a Zeb1-dependent manner. **a**, Relative viability and death rate in MDA-MB-231 cells pretreated with the SCD inhibitors MF438 or CAY10566 (both 5 µM, 24 h) before ML210 treatment (72 h). Death rate was measured using 0.6 µM *n* = 3 independent experiments; ordinary two-way ANOVA. **b**, Proportion of SFAs, MUFAs and PUFAs in PE or PI, the proportion of PUFAs in PE and the relative abundance of PI(18:1/18:1) in MDA-MB-231 (shCtrl and shZeb1) cells treated with vehicle (Veh) or CAY10566 (3 µM) for 48 h. *n* = 5 (shCtrl) and *n* = 4 (shZeb1) from independent experiments; two-tailed paired Student’s *t*-test or ordinary two-way ANOVA. Exemplary extracted UPLC–MS/MS chromatogram for oxidized PE(18:0_20:4 + 3[O]) species in MDA-MB-231 (shCtrl) cells that were pretreated with Veh or CAY10566 (3 µM, 48 h), followed by Veh or RSL3 (1 µM, 2 h) for 2 h. cps, counts per second. **c**, Relative cell viability and cell death rate in MDA-MB-231 cells pretreated with Veh (dimethylsulfoxide (DMSO)/ethanol), 500 µM oleic acid and/or 5 µM MF438, followed by ML210 for 72 h. Cell death was monitored using 0.4 µM ML210 *n* = 3 independent experiments, ordinary two-way ANOVA. **d**, Relative cell viability in MDA-MB-231 (shCtrl and shZeb1) cells pretreated with MF438 (5 µM) for 24 h, followed by 72 h ML210 *n* = 3 independent experiments, ordinary two-way ANOVA. **e**, Growth of fluorescently labelled MDA-MB-231 wild-type (WT) or shZeb1 cells, pretreated with 5 µM MF438 or DMSO as control, in precision-cut tissue slices of mouse lungs. Co-cultures were treated with 0.5 µM ML210 ± 1 µM Fer-1 and surviving tumour cells were quantified 4 days later. *n* = 4 (WT) and *n* = 3 (shZeb1); one-way ANOVA. **f**, Relative cell viability and cell death rate in MDA-MB-231 (shCtrl and shZeb1) cells pretreated with 10 µM of 20:4 (arachidonic acid, AA) for 24 h, followed by 40 h ML210 treatment. Death rate was monitored using 1 µM ML210. *n* = 3 independent experiments; ordinary two-way ANOVA. **g**, Number and occupied area of lung metastases in mice analysed 3 weeks after intravenous injection of KPCmix cells pretreated for 12 h with 10 µM AA, 500 µM oleic acid or ethanol as control. *n* = 27 (9 mice per treatment, from *n* = 3 independent experiments); Kruskal–Wallis test with multiple comparisons. **h**, Relative cell viability and cell death rate in MDA-MB-231 cells pretreated with the FADS2 inhibitor sc-26196 (10 µM) for 24 h, followed by 72 h ML210 treatment. Death rate was measured using 0.37 µM ML210, *n* = 3 independent experiments; ordinary two-way ANOVA. **i**, Relative cell death rate in MDA-MB-231 cells pretreated with vehicle (Ctrl), 10 µM AA and/or 10 µM sc-26196 for 24 h before adding ML210 (3 µM) monitored for 20 h. *n* = 3 independent experiments; ordinary two-way ANOVA. **j**, Cell viability of MDA-MB-231 (shCtrl and shZeb1) cells treated with increasing doses of ML210 after pretreatment with the FADS2 inhibitor sc-26196 (10 µM) for 24 h. *n* = 3 independent experiments; ordinary two-way ANOVA. Data are mean ± s.e.m.

**Extended Data Fig. 6:**
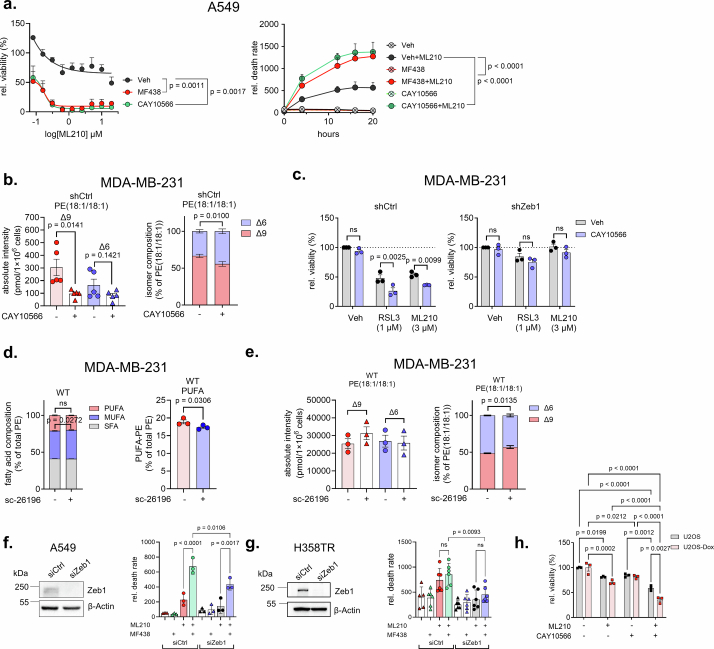
Manipulation of crucial enzymes adjusting the PUFA/MUFA ratio affects ferroptosis sensitivity. **a**) Relative viability and death rate in A549 cells treated with 5 µM of the SCD inhibitors CAY10566 or MF438 for 24 h, followed by ML210 treatment for 72 h. Death rate is displayed for 24 h at 0.6 µM ML210. n = 3 independent experiments, ordinary two-way ANOVA. **b**) Absolute amounts (pmol/1×106 cells) and relative abundances (% of total phosphatidylethanolamine, PE) of PE(18:1/18:1) isomers (Δ9: SCD1-specific isomer; Δ6: FADS2-specific isomer) in MDA-MB-231 (shCtrl and shZeb1) cells treated with vehicle or the SCD inhibitor CAY10566 (3 µM) for 48 h. n = 5 independent experiments, two-tailed paired student t-test or ordinary two-way ANOVA. **c**) Relative viability of MDA-MB-231 (shCtrl and shZeb1) cells treated with vehicle (Veh), the GPX4 inhibitors RSL3 (1 µM) or ML210 (3 µM) in the absence (Veh) or presence of CAY10566 (3 µM) for 48 h. n = 3 independent experiments, repeated measures two-way ANOVA. **d, e**) Proportions of saturated (SFA), monounsaturated (MUFA) and polyunsaturated fatty acids (PUFA) in PE, the proportion of PUFA in PE, and absolute amounts (pmol/1×106 cells) and relative abundances (% of total PE) of PE(18:1/18:1) isomers (Δ9: SCD1-specific isomer; Δ6: FADS2-specific isomer) in MDA-MB-231 (WT) cells treated with sc-26196 (10 µM) for 24 h. n = 3 independent experiments, two-tailed paired student t-test. **f, g**) Immunoblots and SCD inhibition-induced ferroptosis sensitization after siRNA mediated depletion of Zeb1 in A549 (f) and H358TR (g) cancer cells. Bar graphs show relative death rates of cells pretreated with DMSO or 5 µM MF438, followed by ML210 treatment (0.3 µM for A549, 0.63 µM for H358TR) for 16 h. n = 3 independent experiments, ordinary two-way ANOVA. **h**) Relative viability of U2OS and doxorubicin-resistant U2OS (U2OS-Dox) cells treated with ML210 (10 µM), the SCD inhibitor CAY10566 (10 µM) or the indicated combinations for 48 h. n = 3 independent experiments, ordinary two-way ANOVA. All data are presented as presented as mean ± SEM.

Pharmacological inhibition of SCD increased ferroptosis sensitivity in MDA-MB-231 (Fig. 5a) and A549 cells (Extended Data Fig. 6a). SCD inhibition resulted in only a slight, but significant increase in the phospholipid PUFA:MUFA ratio and a higher level of phospholipids with (per)oxidized PUFAs (PE(18:0_20:4)). Moreover, it led to a strong reduction in the cyto-protective lipokine PI(18:1/18:1) (Fig. 5b), matching the activity of SCD to produce oleic acid (Extended Data Fig. 4a). Consequently, the pro-ferroptotic effect of SCD inhibition could be fully reversed by the exogenous supplementation with this MUFA (Fig. 5c). The selectivity for SCD inhibition was demonstrated by its stronger inhibitory effect on the SCD-specific marker PE(18:1Δ9/18:1Δ9) versus the Δ6 isomer (Extended Data Fig. 6b). In contrast to Zeb1^high^ cells, Zeb1-depleted cells did not enhance ferroptosis sensitivity upon SCD inhibition (Fig. 5d and Extended Data Fig. 6c), although SCD activity was efficiently reduced, as indicated by the reduction of the SCD-dependent marker PI(18:1/18:1) in both cell states (Fig. 5b). The results could be confirmed in an ex vivo mouse model, where tumours from implanted cells grew in the tissue context of precision-cut lung slices, and only for wild-type Zeb1, but not for Zeb1-depleted cells, SCD inhibition cooperated with ML210 to reduce tumour growth by ferroptosis (Fig. 5e). To test whether the failure of SCD inhibition to sensitize Zeb1-depleted cells to GPX4 inhibition was due to general ferroptosis resistance, we supplemented arachidonic acid as a pro-ferroptotic PUFA. Arachidonic acid further increased ferroptosis sensitivity in MDA-MB-231 cells, but this was less in Zeb1-depleted clones (Fig. 5f). This shows that Zeb1 expression remains a critical determinant for overall ferroptosis sensitivity and is crucial for additional steps of ferroptotic cell death. Manipulating only one of them (PUFA:MUFA ratio by SCD inhibition or direct addition of PUFAs) is not sufficient to restore ferroptosis sensitivity. The regulation of ASCL4 expression by Zeb1 is probably one of the underlying effects. The results generated in vitro could be partially validated in mouse models by tail vein injection of cancer cells pretreated with either pro-ferroptotic arachidonic acid or anti-ferroptotic oleic acid. Here, arachidonic acid treatment decreased, and oleic acid treatment increased, subsequent formation of lung metastasis (Fig. 5g).

In contrast to SCD manipulation, selective inhibition of FADS2 decreased ferroptosis sensitivity (Fig. 5h) and slightly reduced the proportion of PUFAs in phospholipids (Extended Data Fig. 6d). The selectivity for FADS2 inhibition was demonstrated by a reduction of the FADS2 product 18:1Δ6 versus the SCD product 18:1Δ9 in PE(18:1/18:1) (Extended Data Fig. 6e). The anti-ferroptotic effect of FADS2 inhibition was partially compensated by addition of arachidonic acid (Fig. 5i), whose biosynthesis is dependent on FADS2^27^. As already demonstrated for SCD inhibition, the ferroptosis modulating effect also relied on Zeb1 expression, as FADS2 inhibition had no anti-ferroptotic effect on the low ferroptosis sensitivity in Zeb1-depleted cells (Fig. 5j).

In summary, the inhibition of Zeb1-regulated enzymes that control the synthesis of relevant MUFAs and PUFAs affects ferroptosis sensitivity. These effects are partially dependent on the presence of Zeb1.

### SCD inhibitors sensitize to ferroptosis in translationally relevant settings

Most common human carcinomas show a more or less differentiated epithelial phenotype, and many of them are initially sensitive to standard chemotherapy. However, cancer cells of those tumours often exhibit a high phenotypic plasticity and can respond to external stimuli, for example from the changing tumour environment or to applied chemotherapy, resulting in the acquisition of a transient and partial mesenchymal state^3^. As shown before, this state also confers resistance to standard therapy, but gains high susceptibility to ferroptosis. These observations suggest translational relevance and clinical options for pharmacological intervention.

**Fig. 6:**
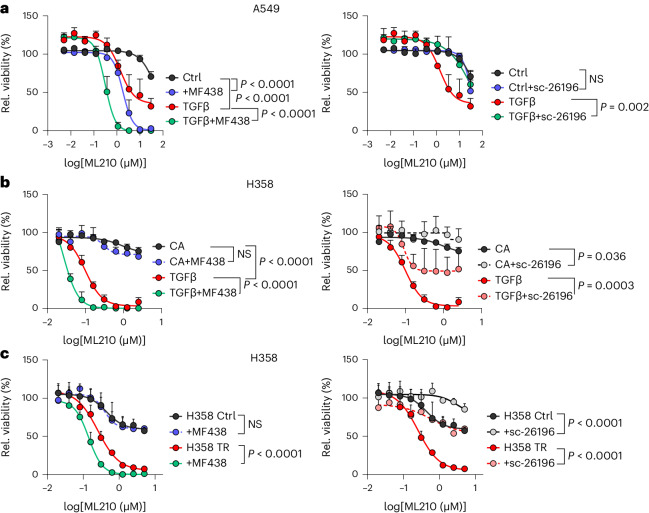
SCD inhibitors sensitize to ferroptosis in translationally relevant settings. **a**,**b**, Reciprocal effect of SCD1 inhibition and FADS2 inhibition on TGFβ-induced ferroptosis sensitivity in A549 and H358 lung carcinoma cells. Relative cell viability of A549 (**a**) and H358 (**b**) cells that were stimulated with 5 ng ml^−1^ TGFβ for 4 and 9 days, respectively, then pretreated with 5 µM MF438 (left) or 10 µM sc-26196 (right) for 24 h, and exposed to the indicated concentrations of ML210 for 48 h (H358) or 72 h (A549). Data are presented as mean ± s.e.m. from *n* = 3 independent experiments; ordinary two-way ANOVA. **c**, Relative cell viability of control and erlotinib-resistant (TR) H358 cells treated with 5 µM MF438 (left) or 10 µM sc-26196 (right) for 24 h, followed by ML210 treatment for 72 h. Data are mean ± s.e.m. from *n* = 3 independent experiments; ordinary two-way ANOVA. NS, not significant.

We mimicked the relevant cancer cell plasticity by analysing the effects of lipogenic enzyme inhibition in therapy resistance models as well as after triggering a partial mesenchymal transition with TGFβ. Inhibition of SCD in A549 and H358 lung cancer cells further boosted the TGFβ-induced ferroptosis sensitivity (Fig. 6a,b). As expected, inhibition of FADS2 had the opposite effect and attenuated TGFβ-induced ferroptosis sensitivity (Fig. 6a,b). Notably, SCD inhibition did not sensitize H358 cells to ferroptosis, when they were not exposed to TGFβ (Fig. 6b) and therefore expressed very low levels of Zeb1 (Extended Data Fig. 1c). However, ferroptosis sensitivity could be further enhanced by SCD inhibition upon TGFβ stimulation (Fig. 6b), which led to a Zeb1^high^ phenotype (Extended Data Fig. 1c). This contrasts with A549 lung cancer cells, which constitutively express higher levels of Zeb1 (Extended Data Fig. 1c), and in which SCD inhibition already has a pro-ferroptotic effect without TGFβ stimulation (Fig. 6a). Notably, in contrast to H358 wild-type cells, SCD inhibition also enhanced ferroptosis sensitivity in erlotinib-resistant H358 cells (Fig. 6c), which acquired a Zeb1^high^ mesenchymal phenotype (Fig. 1f). Again, the cooperative effect of SCD inhibition on ferroptosis activation by ML210 was significantly lower in A549 and H358TR when Zeb1 was depleted (Extended Data Fig. 6f,g). As an additional resistance model, we used the human osteosarcoma cell line U2OS with constitutive Zeb1 expression. Doxorubicin-resistant U2OS variants showed a slightly increased ferroptosis susceptibility. SCD inhibition further increased ferroptosis sensitivity, with the effect being significantly stronger in doxorubicin-resistant cells (Extended Data Fig. 6h).

These data indicate that SCD inhibitors can be used to further sensitize highly aggressive and often therapy-resistant cancer cells in a (partial) mesenchymal state (Zeb1^high^) to ferroptosis-inducing drugs.

## Discussion

A mesenchymal cell phenotype is associated with increased ferroptosis susceptibility^6^. Here, we exemplify an important role of the EMT-TF Zeb1 in ferroptosis by describing one of the mechanisms that render cells vulnerable to phospholipid peroxidation. Zeb1 is a transcriptional regulator, which, depending on the genomic context, can repress or activate target gene transcription^28,34^. We show that Zeb1 represses the expression of enzymes important for MUFA-biosynthesis (*SCD* and *FASN*) and in parallel activates the expression of enzymes central for pro-ferroptotic PUFA production (*FADS2* and *ELOVL5*) and incorporation of PUFAs into phospholipids (*ACSL4*). In particular, ACSL4, which is central for PUFA incorporation, was shown to be crucial for ferroptosis^30^.

What could be the biological relevance of a link between the EMT-TF Zeb1 and the regulation of PUFA phospholipid abundance? Compared with MUFAs, the incorporation of PUFAs into phospholipids facilitates membrane fluidity^35,36^, a prerequisite for enhanced motility of mesenchymal versus epithelial cells. Thus, it is likely that remodelling the cell membrane is a physiological part of the EMT programme, and like other changes, for example in cell polarity, is regulated by EMT-TFs. This is supported by the fact that in our model systems, the increased ferroptosis sensitivity was coupled to induction of a mesenchymal phenotype, which was dependent on Zeb1, but not on the EMT-TFs Snail and Twist1. The latter finding also fits the proposed non-redundant functions of various EMT-TFs^37^. Accordingly, the evolutionary development of the EMT programme made it necessary to co-develop ferroptosis-protective systems in mesenchymal cell types. In other words, a Zeb1-induced, tumour progression-favouring mesenchymal state, which includes abundant incorporation of PUFAs into cancer cell membranes, comes with the price of an associated vulnerability—the high sensitivity to ferroptosis. Thus, inhibition of ferroptosis protection systems—for example, by GPX4 inhibitors—is particularly effective in highly aggressive mesenchymal-type cancer cells.

Owing to the well-established tumour-promoting effects of Zeb1, our findings are of high tumour-biological and translational relevance. Intrinsically high Zeb1 expression characterizes many types of undifferentiated carcinomas and studies demonstrated the association of such tumours with high abundance of PUFA-containing phospholipids and respective enzymes^38–42^. Even more clinically relevant are the common carcinomas with a differentiated phenotype, which can undergo a transient de-differentiation. Particularly, transient Zeb1 expression has been shown to be important for high cancer cell plasticity^15^, coupling high metastatic capacity with high therapy resistance^1–3^, thereby making these cancer cell populations the ultimate therapeutic target. In this context, it is also important that the therapy resistance-associated mesenchymal state gains increased ferroptosis sensitivity. Notably, the Zeb1-dependent pro-ferroptotic effect was also induced by TGFβ, the most prominent example of a tumour-environmental EMT activator, whose temporal and spatial availability triggers cancer cell plasticity^2^. Thus, the environmentally induced, Zeb1-associated cancer cell plasticity as a driver of tumour progression also opens a therapeutic window. As Zeb1 mechanistically links the mesenchymal state-associated tumour-promoting effects with high ferroptosis sensitivity, our data indicate a therapeutic vulnerability of these highly plastic, pro-metastatic and therapy-resistant cancer cells. An indirect support of this view comes from our findings that high expression of FADS2 in undifferentiated tumours (high-grade, mesenchymal subtype) correlates with better survival and particularly distant-metastasis-free survival (Fig. 4b).

To further explore the translational aspects, we focused on the lipogenic enzymes SCD and FADS2, for which pharmacological inhibitors are available and, in the case of SCD, have entered clinical trials^43^. Confirming previous data^44,45^, we show that SCD inhibitors increased ferroptosis sensitivity. However, we here describe that the synergistic pro-ferroptotic effect of SCD and GPX4 inhibitors is stronger in high Zeb1-expressing cell states. Accordingly, Zeb1 expression in tumour cells might also be a useful predictive marker for future combination treatments inducing ferroptosis in highly aggressive cancers. The pro-ferroptotic effect of SCD inhibition is probably also due to the reduced synthesis of MUFAs^46^ and may be enhanced by reduced levels of the cyto-protective lipokine PI(18:1/18:1)^12^. Of note, also additional functions of SCD, for example in maintaining a cancer stem cell state^47^ and in the general cancer cell plasticity between a de-differentiated and differentiated state may be involved^48,49^. In addition, as MUFAs are critical for cell growth, highly proliferating cancer cells depend on SCD expression and its pharmacological inhibition reduces cancer cell growth^43^. Moreover, due to the dependency of cancer cells on SCD and other lipogenic enzymes, these factors are not only regulated by cell intrinsic determinants, but also by the specific conditions of the tumour environment, for example the availability of oxygen and external sources of unsaturated lipids^50,51^. This was shown for Her2^+^ breast cancer brain metastasis, where the low-fatty-acid environment leads to SCD and FASN dependency, which subsequently results in an increased sensitivity to SCD inhibitors^52,53^. The opposite has been shown for inhibition of FADS2, which is expressed in various undifferentiated cancer types^54^, and supports ferroptosis through stimulating PUFA synthesis. FADS2 inhibitors attenuate ferroptosis, indicating potential therapeutic strategies for diseases with increased ferroptosis sensitivity, for example neurodegenerative diseases^13^. Together, owing to their pleiotropic homoeostatic functions, the regulation of lipogenic enzyme expression is complex and Zeb1 adds one level of complexity in the course of EMT-associated plasticity.

In summary, we describe that Zeb1-controlled EMT and cellular plasticity involves a metabolic reprogramming, including the regulation of the PUFA:MUFA ratio in phospholipids, which is critical for ferroptosis sensitivity. Thereby, ACSL4, ELOVL5, FADS2, SCD and FASN are counteracting Zeb1 downstream targets. Our data are of potential translational relevance and suggest a combination of SCD inhibitors and ferroptosis activators for the treatment of aggressive cancers expressing high Zeb1.

## Methods

### Animal ethics statement

Animal husbandry and experiments were approved by the committee of ethics of animal experiments of the state of Bavaria (Regierung Unterfranken, Würzburg; Regierung von Unterfranken, Würzburg; TS-30-2021, 55.2DMS2532-2-1832) and performed according to the European Animal Welfare laws and guidelines. Mice were kept on a 12-h light–dark cycle in individually ventilated cages at a constant temperature between 20–24 °C and 45–65% humidity and provided with food and water ad libitum in the animal facilities of the Friedrich-Alexander University of Erlangen-Nürnberg.

### Cell lines and cell culture

MDA-MB-231, MCF7, H358, A549, BxPC3, 143B and U2OS cells were purchased from the American Type Culture Collection. Generation of various mouse KPC cell lines from KPC tumours is previously described^15^. They were cultured under standard conditions at 37 °C and 5% CO_2_ in DMEM (Gibco) supplemented with 10% foetal bovine serum (Gibco) and regularly tested for *Mycoplasma* contamination. Generation of 143BshZeb1 clones is previously described^55^. MDA-MB-231 shCtrl and shZeb1 cells^56^ were cultivated in the presence of puromycin (1 µg ml^−1^) for 7 days every 1 month to maintain stable transfection. CRISPR–Cas9-mediated knockout of EMT-TFs in KPC cells was carried out as previously described^57^. In brief, the sgRNAs targeting *Zeb1* exon 2 (5′-GACCAGACAGTATTACCAGG-3′), *Snai1* exon 1 (5′-GAGCTGCAGGACGCGTGTGT-3′) and *Twist1* exon 1 (5′-CGGGAGCCCGCAGTCGTACG-3′) were cloned into pX459 (Addgene, 62988) and transiently transfected into KPC661 with Lipofectamine 3000, followed by selection with 4 µg ml^−1^ puromycin for 3 days and clonal expansion using FACS. Sequence-verified clones with biallelic indel mutations and protein loss were used. To induce a transient knockdown, cells were transfected with Silencer select siRNAs (Ambion; s229970 for siZeb1, 4390844 for siCtrl) at a final concentration of 50 nM, using Lipofectamine RNAiMAX transfection reagent (Thermo Fisher, 13778) according to the manufacturer’s instructions and cells were treated with indicated ferroptosis inducers 48 h after transfection.

H358 and BxPC3 drug-resistant cell lines were previously described^17,58^ and routinely maintained in 10 µM erlotinib or 40 nM gemcitabine, respectively. Doxorubicin-resistant U2OS cells (U2OS-Dox) were established by continuous treatment of parental cells (at 80% confluence) by stepwise increasing the concentration of doxorubicin (3.75–500 nM; Szabo Scandic) every 2 weeks over 4 months and maintained in 500 nM doxorubicin.

For TGFβ treatment, the medium was supplemented daily with 5 ng ml^−1^ TGFβ1 (PeproTech) for the indicated amount of time as specified in figure legends. As TGFβ1 was dissolved in a citric acid solution, the medium of control cells was supplemented with citric acid to a concentration of 500 nM.

### Chemicals

3-(4,5-Dimethylthiazol-2-yl)-2,5-diphenyltetrazolium bromide, erlotinib, gemcitabine and arachidonic acid were obtained from Sigma-Aldrich. CAY10566, erastin, etoposide, ferrostatin-1, ML210, (1*S*,3*R*)-RSL3, sc-26196 and oleic acid were obtained from Cayman Chemical. MF438 and ferrostatin-1 were from Med Chem Express. 1,2-Dimyristoyl-*sn*-glycero-3-phosphatidylcholine (DMPC), 1-pentadecanoyl-2-oleoyl(d7)-*sn*-glycero-3-phosphocholine (PC(15:0/18:1-d7)), 1,2-dimyristoyl-*sn*-glycero-3-phosphatidylethanolamine (DMPE), 1-pentadecanoyl-2-oleoyl(d7)-*sn*-glycero-3-phosphoethanolamine (PE(15:0/18:1-d7)), 1-pentadecanoyl-2-oleoyl(d7)-*sn*-glycero-3-phosphoinositol (PI(15:0/18:1-d7)), PE(16:0/20:4) and oxPC(16:0/20:4) (oxPAPC) were obtained from Avanti Polar Lipids, dissolved in chloroform, aliquoted and stored under argon and protected from light at −80 °C.

### Plasmids

The generation of the LLGL2 and ANKRD1 luciferase reporter plasmids was previously described^28,56^. The promoter luciferase reporter plasmids were generated by amplifying the FADS2 promoter (−619 to +233 rel. to transcription start site (TSS)), the ACSL4 (−532 to +1008 rel. to TSS), the FASN promoter (−1305 to +196 rel. to TSS) and the SCD promoter (−976 to +69 rel. to TSS) from genomic DNA by PCR. The restriction sites XhoI and BglII were incorporated into the primers (Supplementary Table 1) and the amplicons were inserted into pGL4.10 (E6651, Promega). For the ELOVL5 luciferase reporter vector, the intronic region chr6: 53,326,815–53,325,856 (hg38) was amplified from genomic DNA. Restriction enzyme sites XhoI and BglII were incorporated into the primers and the amplicon was inserted into pGL4.23 (E8411, Promega). pCIneo-hZEB1 was a gift from M. M. Sanders (University of Minnesota).

### Luciferase reporter assay

MCF7 cells were seeded in 24-well plates in triplicate at 20% density. The next day, they were transfected with the FuGENE HD transfection reagent (Promega, E2311) according to the manufacturer’s instructions, using 100 ng firefly luciferase reporter vector and 30 ng pRL-TK Renilla luciferase control reporter vector (Promega, E2241) together with 100 ng ZEB1 expression vector or the corresponding empty control vector, respectively. Cells were collected after 72 h and lysed in passive lysis buffer (Promega, E1941). Luciferase activity was measured using the Dual-Luciferase Reporter Assay system and a CentroXS^3^ LB 960 Luminometer (Berthold). Values of the firefly luciferase were normalized to their corresponding Renilla values, serving as a transfection control.

### Chromatin profiling

ATAC-seq and ChIP–seq for ZEB1 were performed as described previously^28^. ChIP–seq for H3K27ac (rabbit anti-trimethyl histone H3K27ac, Millipore 07-449) was performed accordingly using MDA-MB-231 shCtrl or shZEB1 cells, except that the EGS crosslinking step was omitted. Crosslinking with 1% formaldehyde was performed for 5 min directly on the plate in cell growth medium.

### Western blot analysis

For the analysis of whole cell protein, cells at 50–70% density were lysed in ice-cold lysis buffer (150 mM NaCl, 50 mM Tris-HCl, pH 8.0, 0.5% sodium deoxycholate (*w*/*v*), 0.1% SDS (*v*/*v*), 1% NP40 (*v*/*v*), 1 mM PMSF, 1× complete protease inhibitor cocktail (Roche, 04693132001) and 1× PhosphoStop (Roche, 4906837001)). Protein concentration was determined using the BCA Protein Assay (Thermo Fisher Scientific, 23225) according to the manufacturer’s instructions. Protein samples were separated by SDS–PAGE, followed by wet blot transfer onto nitrocellulose membranes (Roth, 4685.1). Primary antibodies were applied overnight at 4 °C and secondary antibodies were applied for 1 h at room temperature (RT). For protein detection Western Lightning Plus ECL solution (Perkin-Elmer, NEL105001EA) and the ChemiDoc MP Imaging System (Bio-Rad) with their respective software, ImageLab 6.1, were used. Western blot band quantification was performed using ImageJ v.153a. The following antibodies were used: rabbit anti-ZEB1 (1:2,000 dilution, HPA027524, Sigma-Aldrich), mouse anti-E-cadherin (1:5,000 dilution, 610182, BD Transduction Laboratories), mouse anti-β-actin (1:10,000 dilution, A5441, Sigma-Aldrich), rabbit anti-SCD (1:1,000 dilution, 23393-1-AP, Proteintech), rabbit anti-FASN (1:1,000 dilution, MA5-14887, Thermo), rabbit anti-FADS2 (1:1,000 dilution, 28034-1-AP, Proteintech) and rabbit anti-ELOVL5 (1:1,000 dilution, PA583879, Thermo), mouse anti-ACSL4 (1:1,000 dilution, Santa Cruz, sc-271800), rabbit anti-GAPDH (1:10,000 dilution, Cell Signalling, 2118), mouse anti-SNAIL (1:500, Cell Signalling, 3895), rabbit anti-TWIST (1:1,000 dilution, Abcam, ab50581), goat anti-Mouse IgG Peroxidase (1:10,000 dilution, 115-035-1463, Jackson ImmunoResearch); and goat anti-rabbit IgG peroxidase (1:10,000 dilution, 111-035-144, Jackson ImmunoResearch).

### Immunofluorescence and image acquisition

For immunofluorescence labelling, cells seeded onto sterile glass coverslips were fixed in 4% PFA, quenched and permeabilized in 0.2% Triton X-100/100 mM glycine/PBS, pH 7.4 and blocked in 3% BSA/PBS at room temperature. Primary and secondary antibodies were diluted in blocking solution and incubated for 1 h or 45 min, respectively, at room temperature in a humidified chamber protected from light. Nuclei were stained with DAPI (Sigma, D9542) before coverslips were mounted onto glass slides with CitiFluor AF1 solution (EMS, 17970-100). Images were acquired using a Leica DM5500 B microscope and processed using the Leica Application Suite X software. The following antibodies were used: rabbit anti-ZEB1 (1:250 dilution, HPA027524, Sigma-Aldrich), rabbit anti-vimentin (1:250 dilution, 5741, Cell Signalling), mouse anti-E-cadherin (1:250 dilution, 610182, BD Transduction Laboratories), Alexa Fluor 555 goat anti-rabbit IgG (H+L) (1:300 dilution, A21428, Life Technologies); Alexa Fluor 488 goat anti-mouse IgG (H+L) (1:300 dilution, A11029, Life Technologies).

### Cell viability and death assay

To determine cell viability, cells were plated at 5–10% confluence in 96-well plates. After 16–24 h, cells were treated with vehicle or the indicated compounds at the given concentrations for 48–72 h. DMSO served as solvent control for most compounds, with the exception of ethanol (used for arachidonic acid and oleic acid) and PBS (used for gemcitabine). To determine the sensitization to ferroptosis by SCD and FADS2 inhibition, cells were pretreated with 5 µM MF438, 3 or 5 µM CAY10566 or 10 µM sc-26196 for the indicated time before the addition of ferroptosis inducers. To investigate the effect of exogenous fatty acid supplementation, cells were incubated with 10 µM 20:4/arachidonic acid or 500 µM 18:1/oleic acid for 12–16 h before ML210 was added. Ferroptosis was inhibited by co-treatment with ferrostatin-1 at a final concentration of 1 µM. Cell viability was either assessed by measuring cellular dehydrogenase activity via an MTT assay according to the manufacturer’s instructions or the confluence matrix using the live-cell imaging device Incucyte S3 (Sartorius). Confluence or absorbance data for the individual treatments were subtracted at the indicated time points either from their initial values or from positive controls, normalized to the mean value of control cells or the individual controls (each set to 100%) and optionally plotted to their respective, log-transformed drug concentrations. Cytotoxicity was measured using SYTOX Green nucleic acid stain (Thermo Fisher Scientific) at a final concentration of 5 nM. The cell death rate was calculated by normalizing the number of SYTOX Green-positive objects, indicating dead cells, to cell confluence (%) for each time point and condition.

### Lung colonization

KPC(mix) cells were pretreated for 12 h with 10 µM 20:4/arachidonic acid, 500 µM 18:1/oleic acid or ethanol as vehicle control. Subsequently, 100 μl PBS containing 5 × 10^4^ cells were injected into the tail vein of 8-week-old C57BL/6NRj mice (Janvier Labs). Littermates of both sexes were randomized for all treatment cohorts, monitored twice per week and killed 3 weeks after injection. Lungs were isolated, fixed in 4% paraformaldehyde and embedded in paraffin. Lung tissues were sectioned at 4-µM thickness and stained with haematoxylin and eosin solution. Per mice, metastatic lesions were screened on three sections separated by at least 200 μm. Quantification was performed by analysing the number of metastases as well as metastatic areas normalized to the respective lung area using ImageJ v.1.53a. For each treatment condition, nine mice were used in three independent experiments. The number and size of metastases never exceeded the maximal burden permitted by the local authorities.

### Precision-cut lung slices

Precision-cut lung slices (PCLSs) were obtained from 8-week-old female C57BL/6NRj mice (Jackson Laboratory) using a vibratome VT1200S (Leica). On the same day, fluorescently labelled MDA-MB-231 wild-type (mCherry) or shZeb1 (tdTomato) were pretreated with 5 µM MF438 or DMSO as vehicle control. The following day, single lung slices were incubated with 1 × 10^5^ cells in low-adherent 48-well plates for 4 h. After transfer into fresh plates, PCLS tumour cell co-cultures were treated with 0.5 µM ML210 ± 1 µM ferrostatin-1 or DMSO as control. Imaging was performed using the EVOS system (M7000 Thermo Fisher) before and 4 days after treatment. Fluorescent signals were quantified and normalized to their respective PCLS areas using ImageJ v.1.53a. The resulting values from day 4 were divided by those from day 0, followed by normalization to their respective DMSO controls. PCLS viability was confirmed using CyQUANT LDH Cytotoxicity Assay (Invitrogen).

### Analyses of mouse allograft tumours

Cryo-conserved tumour specimens were obtained from subcutaneous allografts described by Krebs et al.^15^. We analysed two tumours derived from mesenchymal KPC cell lines (KPC550 and KPC701) and epithelial and mixed KPC cell lines (KPC438, KPC661 and 792) (with wild-type Zeb1 or heterozygous alleles), as well as one tumour from the KPCZ-derived cell lines (346, 387, 426 and 519) with Zeb1-knockout alleles for protein expression by immunohistochemistry (IHC) and for abundance of phospholipids by UPLC–MS/MS. For IHC, serial sections (4 µm) were treated as described^15^. The following primary antibodies were used: polyclonal rabbit anti-Zeb1 (Novus Biologicals, NBP1-05987, diluted 1:150), polyclonal rabbit anti-FADS2 (Proteintech, 28034-1-AP, diluted 1:100), polyclonal rabbit anti-ELOVL5 (Novus Biologicals, NBP3-14304, diluted 1:50), monoclonal rabbit anti-FASN (Thermo Fisher Scientific, MA5-14887, diluted 1:50), polyclonal rabbit anti-SCD (Proteintech, 23393-1-AP, diluted 1:250), polyclonal rabbit anti-ACSL4 (Santa Cruz, sc365230, diluted 1:250) and polyclonal rabbit anti-4-HNE (Abcam, ab46545, diluted 1:250). Tumours were grouped and analysed in three different ways according to (1) Zeb1 expression (IHC score ≤1 versus >1); (2) Zeb1 genotype (wild type/KPC versus Zeb1 knockout/KPCZ); and (3) histological tumour grade (grade ≤2 versus >2). IHC expression in the tumour cells was scored from 0 (low) to 3 (high). For UPLC–MS a ~30–50-mg fresh-frozen cryo-conserved tumour sample was used for the procedure described in ‘Extraction and analysis of phospholipids’.

### Zebrafish engraftment

Zebrafish larvae were injected as previously described^59,60^. In brief, 300 tdTomato-positive MDA-MB-231 shCtrl or shZeb1 cells, resuspended in 2% polyvinylpyrrolidone 40 (PVP40, Sigma)/DPBS were injected intravenously via the duct of Cuvier of Tg(fli:eGFP) × casper zebrafish larvae at 48 h after fertilization using capillary glass needles. For ferroptosis rescue experiments, cells were pretreated with Fer-1 24 h and 4 h before transplantation. Engraftment procedures have been previously described^10^. Engrafted individuals were imaged at 1 and 3 days after implantation using an epifluorescence stereo microscope. All images were analysed using a custom ImageJ MACRO. Data were normalized to the wild-type control group of each experimental population or, in the case of drug treatments, to the vehicle control group; two biological replicates were combined with at least 20 individuals per biological replicate.

### RNA extraction and qRT–PCR

Total RNA was isolated and reversely transcribed using the RNeasy Plus Mini kit (QIAGEN, 74136) and the RevertAid First Strand cDNA Synthesis Kit (Thermo Fisher Scientific, K1622) according to the manufacturer’s instructions. cDNA was amplified in 384-well plates using gene-specific primers (Supplementary Table 2), with Power SYBR Green PCR Master Mix (Applied Biosystems, 4367659) according to the manufacturer’s protocol. Samples were run in triplicates in a LightCycler 480 (Roche) and normalized to *GAPDH*, *ACTB* or *HPRT1*.

### Gene Ontology analysis

The target genes list of Zeb1-bound promoters was obtained from Feldker et al.^28^, and the differentially expressed genes between the KPC wild-type and KPC Zeb1-knockout conditions^1^ were used to perform Gene Ontology term analysis using ClusterProfiler R package (v.4.0)^11,61^. Ensemble Gene symbols were fed into the tool with the default settings and the top 20 biological processes were selected for plotting.

### Motif analysis

Analysis of transcription factor binding motifs was performed using LASAGNA-Search: an integrated web tool for transcription factor binding site search and visualization^62^.

### Extraction and analysis of phospholipids

Lipids were extracted from cell pellets or tumour allografts by the sequential addition of PBS, methanol, chloroform and saline (at a final ratio of 14:34:35:17)^63,64^. The chloroform layer was recovered, brought to dryness using an Concentrator Plus System (Eppendorf) and the lipid film was dissolved in methanol. Internal standards (0.2 nmol each): Phosphatidylcholine (14:0/14:0), PE(14:0/14:0), PC(15:0/18:1-d7), PE(15:0/18:1-d7) and/or PI(15:0/18:1-d7).

Phospholipids were separated on an Acquity UPLC BEH C8 column (130 Å, 1.7 μm, 2.1 × 100 mm, Waters) using an Acquity UPLC (Waters) coupled to a QTRAP 5500 mass spectrometer (Sciex)^65,66^ or an ExionLC AD UPLC (Sciex) coupled to a QTRAP 6500^+^ mass spectrometer (Sciex), both equipped with an IonDrive Turbo V Ion Source and a TurboIonSpray probe^12^. For the latter, chromatographic separation was performed at 45 °C and 0.75 ml min^−1^ using mobile phase A (water:acetonitrile, 90:10, 2 mM ammonium acetate) and mobile phase B (water:acetonitrile, 5:95, 2 mM ammonium acetate). The gradient was ramped from 75 to 85% B over 5 min and increased to 100% B within 2 min, followed by isocratic elution for 2 min. The MS source and compound parameters for the QTRAP 6500^+^ mass spectrometer are shown in Supplementary Table 3. Phospholipids were analysed in the negative ion mode by multiple reaction monitoring (MRM), and the mean signal of the two fatty acid anion fragments was calculated.

Mass spectra were obtained and processed with Analyst (v.1.6.3 or v.1.7.1)^67^. To calculate absolute PE and phosphatidylinositol quantities, signals were normalized to protein content and a subgroup-specific deuterated internal standard. To calculate relative intensities, all analysed signals within the subgroup were summarized (=100%) and the signals of individual lipids were expressed as percentage of this sum. The fractions of PUFAs, MUFAs and SFAs in phospholipids were calculated from mean signal intensities divided by two and equally distributed to the sn-1 or sn-2 fatty acids. The proportions of PUFA- and non-PUFA-containing phospholipid species in Figs. 2c and 3c and Extended Data Fig. 2e (pie charts) summarize the relative intensities of phospholipids species either containing at least one PUFA or exclusively carrying SFA/MUFA.

### Extraction and analysis of oxidized phospholipids

Oxidized phospholipids were extracted as described above and analysed using an ExionLC AD UHPLC system (Sciex) coupled to a QTRAP 6500^+^ mass spectrometer (Sciex) by MRM in a negative ion mode. The MS source and compound parameters are shown in Supplementary Table 3.

Oxidized phospholipids were identified from the fragments of [M-H]^−^ (oxidized phosphatidylethanolamine, oxidized phosphatidylinositol) or [M+OAc]^−^ (oxidized phosphatidylcholine) ions indicated in Supplementary Table 4. Signals were analysed only when retention times applied to the effective carbon number model and were within predefined ranges (Supplementary Tables 5–7). The definition of retention time windows was supported by reference phospholipids, oxPAPC (Avanti Polar Lipids) and oxidized PE(16:0/20:4). The latter was obtained by enzymatic oxygenation of PE(16:0/20:4) (Avanti Polar Lipids) with a lipoxidase (type V) from glycine max (soybean, L6632; batch no. SLCC4512; Sigma-Aldrich)^68^. The retention time windows for oxPAPC (1[O]: 2.8–4.6 min; 2[O]: 2.9–4.3 min; and 3[O]: 1.45–2.3 min) and oxidized PE(16:0/20:4) (1[O]: 2.79-2.96 min; 2[O]: 2.84-3.04 min) were extended to include potential regioisomers^69^ and adapted to additional phosphatidylcholine, phosphatidylethanolamine or phosphatidylinositol species based on the effective carbon number model, as listed in Supplementary Tables 5–7. Oxidized phospholipids were quantified based on the most intensive, specific transition to the oxidized fatty acid anions. To calculate the amount of phospholipids with one [1O], two [2O] or three oxygens [3O] incorporated, the individual signals within the defined retention time windows were summarized without discriminating between isomers and normalized to DMPC (oxidized phosphatidylcholine, oxidized phosphatidylinositol) or DMPE (oxidized phosphatidylethanolamine) and the cell number.

### Public database queries

For the Cancer Dependency Map dataset (Figs. 1b and 3d), the CERES score for each gene of interest or tumour was downloaded from the DepMap portal (https://depmap.org/portal). The relationship between compound sensitivity and gene expression (Figs. 1b and 3d) was analysed using datasets from the Cancer Therapeutics Response Portal (http://portals.broadinstitute.org/ctrp.v2.1/)^70–72^. The correlation of expression analysis (Extended Data Fig. 4b.) was performed with cBioPortal using expression data from 732 cell lines of solid cancers from the Cancer Cell Line Encyclopedia (https://www.cbioportal.org/study/summary?id=ccle_broad_2019). For transcriptomic analysis of publicly available RNA-seq datasets from patients with breast cancer, processed MET500 RNA-seq samples (https://xenabrowser.net/datapages/?cohort=MET500)^25^ were downloaded from the UCSC Xena Browser, and Ensembl gene IDs were converted to gene symbols using annotations from Ensembl Release 108. Samples in each compendium were then scored for their position in the EM spectrum. In brief, the expression matrix was scaled (mean centred, with s.d. set to 1) and the previously described method^73^ was applied, using the KS gene signature for tumour cells. For GSVA analysis^74^, we used custom gene sets for MUFAs (SCD and FASN), PUFAs (FADS2, ELOVL5 and ACSL4) and the (single-gene) *ZEB1*. After ranking samples by EM score, GSVA was run with the ssGSEA method, and the GSVA scores were visualized with R’s pheatmap package. The regression line for scatter-plots was calculated using a linear model, as implemented in R. For further meta-analysis of published datasets for survival of patients with breast cancer, colon cancer and pancreatic cancer, we used KM-Plotter (http://kmplot.com/analysis)^33^. Settings were ‘all samples’ or as indicated in the figures for each tumour entity and for all analyses: ‘auto select best cut off’, ‘user selected probe sets’ with selection of the recommended probe set, ‘compute median survival’ and ‘censore at threshold’.

### Statistics and reproducibility

Data analysis was performed using GraphPad Prism 9 software and OriginPro 2021 software. For multiple comparisons, ordinary or repeated measures one-way or two-way ANOVA with Dunnett’s, Tukey’s or Sidak’s post hoc tests were applied. For the comparison of two groups, multiple *t*-tests with false discovery rate of 5% using a two-stage linear step-up procedure by Benjamini, Krieger and Yekutieli or two-tailed unpaired *t*-tests were used. *P* values <0.05 were considered statistically significant. Mouse/fish numbers represent the biological replicates. Sample size and replicates are indicated in the figure legends. All experiments presented in the Article were repeated in at least three independent biological replicates. Data are presented as mean or mean ± s.e.m. of *n* observations, where *n* represents the individual number of experiments, unless otherwise specified in the figure legends. Precise *P* values are in the figure legends. No data were excluded from the analyses. The investigators were blinded to allocation during experiments (IHC analyses) and outcome assessment. No statistical methods were used to predetermine sample sizes but our sample sizes are similar to those reported in previous publications^15,17^. Data distribution was assumed to be normal but this was not formally tested.

### Reporting summary

Further information on research design is available in the Nature Portfolio Reporting Summary linked to this article.

## Online content

Any methods, additional references, Nature Portfolio reporting summaries, source data, extended data, supplementary information, acknowledgements, peer review information; details of author contributions and competing interests; and statements of data and code availability are available at 10.1038/s41556-024-01464-1.

## Supplementary information

Reporting Summary

Supplementary Video 1 Primarily epithelial-type cancer cells survive GPX4 inhibition in mixed cell lines (here KPC438 mixed, treated for 66 h with 16 µM ML210) (red arrows indicate epithelial cancer cells and green arrow indicates mesenchymal cancer cells).

Supplementary Tables Supplementary Tables 1–7 containing oligonucleotide sequences and parameters for mass spectrometry.

## Source data

Source Data Fig. 1 All statistical source data, with clearly named tabs for each figure and extended data figure.

Source Data Fig. 2 All unprocessed blots with clearly labelled blots for each item.

## Data Availability

ChIP–seq data are deposited at the Gene Expression Omnibus (GEO) (GSE264671) and the mass spectrometric lipidomics data generated in this study are deposited in the Metabolomics Workbench database^75^ (project ID PR002015; 10.21228/M85238; study IDs: ST003245, ST003256, ST003257 and ST003259). Other databases/datasets used in this study are DepMap portal/CERES score (https://depmap.org/portal)**;** cBioPortal, CCLE (https://www.cbioportal.org/study/summary?id=ccle_broad_2019). Previously published ChIP–seq and ATAC-seq datasets that were re-analysed here are available in the ArrayExpress database at EMBL-EBI under accession no. E-MTAB-8258 (ZEB1 ChIP–seq data) and E-MTAB-8264 (ATAC-seq). Any additional data supporting the findings reported in this paper are available upon reasonable request. Source data are provided with this paper.

## Code availability

Source codes are available on request to the authors.
