# Supplementary material for: Zeb1 mediates EMT/plasticity-associated ferroptosis sensitivity in cancer cells by regulating lipogenic enzyme expression and phospholipid composition
Source: Nat Cell Biol. 2024 Jul 15;26(9):18. doi: 10.1038/s41556-024-01464-1 (PMC11392809; doi:10.1038/s41556-024-01464-1)
Supplement: Supplementary file 1 — Reporting Summary [file 41556_2024_1464_MOESM1_ESM.pdf]

nature portfolio

Corresponding author(s): Thomas Brabletz, Andreas Koeberle

Last updated by author(s): Jun 19, 2024

Reporting Summary

Nature Portfolio wishes to improve the reproducibility of the work that we publish. This form provides structure for consistency and transparency in reporting. For further information on Nature Portfolio policies, see our Editorial Policies and the Editorial Policy Checklist.

Statistics

For all statistical analyses, confirm that the following items are present in the figure legend, table legend, main text, or Methods section.

n/a	Confirmed	
☐	☒	The exact sample size (*n*) for each experimental group/condition, given as a discrete number and unit of measurement
☐	☒	A statement on whether measurements were taken from distinct samples or whether the same sample was measured repeatedly
☐	☒	The statistical test(s) used AND whether they are one- or two-sided<br>*Only common tests should be described solely by name; describe more complex techniques in the Methods section.*
☒	☐	A description of all covariates tested
☐	☒	A description of any assumptions or corrections, such as tests of normality and adjustment for multiple comparisons
☐	☒	A full description of the statistical parameters including central tendency (e.g. means) or other basic estimates (e.g. regression coefficient) AND variation (e.g. standard deviation) or associated estimates of uncertainty (e.g. confidence intervals)
☐	☒	For null hypothesis testing, the test statistic (e.g. *F*, *t*, *r*) with confidence intervals, effect sizes, degrees of freedom and *P* value noted<br>*Give P values as exact values whenever suitable.*
☒	☐	For Bayesian analysis, information on the choice of priors and Markov chain Monte Carlo settings
☒	☐	For hierarchical and complex designs, identification of the appropriate level for tests and full reporting of outcomes
☐	☒	Estimates of effect sizes (e.g. Cohen's *d*, Pearson's *r*), indicating how they were calculated

Our web collection on statistics for biologists contains articles on many of the points above.

Software and code

Policy information about availability of computer code

Data collection	Phospholipid mass spec data were obtained using an Acquity Ultraperformance LC system (Waters) coupled to QTRAP 5500 mass spectrometer (Sciex) equipped with a Turbo V Ion Source and a TurboIonSpray probe for electrospray ionization 12, 13 or an ExionLC AD UHPLC system (Sciex) that was coupled to a QTRAP 6500+ mass spectrometer (Sciex) equipped with an IonDrive Turbo V Ion Source and a TurboIonSpray probe for electrospray ionization as described in the Methods section.

Data analysis	Phospholipid mass spectra were obtained with Analyst 1.6.3 (QTRAP5500, Sciex) or Analyst 1.7.1 (QTRAP6500+, Sciex) and processed with Analyst 1.6.3 (Sciex) as described in the Methods section. Microscopic images were acquired with a Leica DM5500 B microscope and analysed using the Leica Application Suite X software.<br>ATAC-seq and ChIP-seq data were analysed as described in our previous publication by Feldker et al. The EMBO Journal (2020) 39: e103209.<br>IncuCyte S3 live-cell instrument and analysis software module were used to determine cell death and viability. A more detailed description can be found in the methods section.<br>Precision cut lung slices (PCLS) co-cultures were analysed with the EVOS M7000 imaging system (Thermo).<br>ChemiDocTM Imaging System (BioRad), ImageLab 6.1 and ImageJ v1.53a were used for Western blot analysis.<br>Luciferase assays were performed with the CentroXS³ LB 960 Luminometer (Berthold Technologies).<br>LightCycler® 480 II Software 1.5.1 was used for qRT-PCR data analysis.<br>GraphPad Prism 9 and OriginPro 2021 were used for statistical analysis. GO term analysis was performed using ClusterProfiler R package v.4.0.<br>Raw reads were quality filtered and adapter-trimmed using cutadapt v.1.9.1, then aligned to GRCh38 ensemble genome assembly using Bowtie2 v. 2.2.9.<br>BigWig files were generated using deeptools v 2.3.

For manuscripts utilizing custom algorithms or software that are central to the research but not yet described in published literature, software must be made available to editors and reviewers. We strongly encourage code deposition in a community repository (e.g. GitHub). See the Nature Portfolio guidelines for submitting code & software for further information.

Data

Policy information about availability of data

All manuscripts must include a data availability statement. This statement should provide the following information, where applicable:

- Accession codes, unique identifiers, or web links for publicly available datasets
- A description of any restrictions on data availability
- For clinical datasets or third party data, please ensure that the statement adheres to our policy

New sequence data have been deposited at GEO (GSE264671) and the mass spectrometric lipidomics data generated in this study have been deposited in the Metabolomics Workbench database 21 (project id: PR002015; DOI: https://dx.doi.org/10.21228/M85238; study ids: ST003245, ST003256, ST003257, ST003259) to be publicly available as of the date of publication. Other databases/datasets used in this study are: DepMap portal/CERES score (https://depmap.org/portal); cBioPortal/CCLE (https://www.cbioportal.org/study/summary?id=ccle_broad_2019); UCSC Xena Browser/MET500 RNA-seq samples (https://xenabrowser.net/datapages/?cohort=MET500). Source data are provided with this study. Any additional data supporting the findings reported in this paper is available upon reasonable request.

Research involving human participants, their data, or biological material

Policy information about studies with human participants or human data. See also policy information about sex, gender (identity/presentation), and sexual orientation and race, ethnicity and racism.

Reporting on sex and gender	n/a
Reporting on race, ethnicity, or other socially relevant groupings	n/a
Population characteristics	n/a
Recruitment	n/a
Ethics oversight	n/a

Note that full information on the approval of the study protocol must also be provided in the manuscript.

Field-specific reporting

Please select the one below that is the best fit for your research. If you are not sure, read the appropriate sections before making your selection.

☒ Life sciences ☐ Behavioural & social sciences ☐ Ecological, evolutionary & environmental sciences

For a reference copy of the document with all sections, see nature.com/documents/nr-reporting-summary-flat.pdf

Life sciences study design

All studies must disclose on these points even when the disclosure is negative.

Sample size	No statistical method was used to predetermine sample size. Sample sizes were chosen according to our experience as reported in previous publications (15, 17). Sample sizes are indicated in the figure legends.
Data exclusions	No data were excluded.

Replication	All experiments presented in the Article were repeated in at least three independent biological replicates of which all attmpts at replication were successful. Numbers of replicates are indicated in the figure legends.
Randomization	For i.v. injections as well as for subcutaneous allografts as performed and described in Krebs et al. Nature Cell Biology (2017), available mouse cohorts were grouped and randomized for engraftment of different cell lines.
Blinding	The investigators were blinded to allocation during IHC analyses and outcome assessment.

Behavioural & social sciences study design

All studies must disclose on these points even when the disclosure is negative.

Study description	*Briefly describe the study type including whether data are quantitative, qualitative, or mixed-methods (e.g. qualitative cross-sectional, quantitative experimental, mixed-methods case study).*
Research sample	*State the research sample (e.g. Harvard university undergraduates, villagers in rural India) and provide relevant demographic information (e.g. age, sex) and indicate whether the sample is representative. Provide a rationale for the study sample chosen. For studies involving existing datasets, please describe the dataset and source.*
Sampling strategy	*Describe the sampling procedure (e.g. random, snowball, stratified, convenience). Describe the statistical methods that were used to predetermine sample size OR if no sample-size calculation was performed, describe how sample sizes were chosen and provide a rationale for why these sample sizes are sufficient. For qualitative data, please indicate whether data saturation was considered, and what criteria were used to decide that no further sampling was needed.*
Data collection	*Provide details about the data collection procedure, including the instruments or devices used to record the data (e.g. pen and paper, computer, eye tracker, video or audio equipment) whether anyone was present besides the participant(s) and the researcher, and whether the researcher was blind to experimental condition and/or the study hypothesis during data collection.*
Timing	*Indicate the start and stop dates of data collection. If there is a gap between collection periods, state the dates for each sample cohort.*
Data exclusions	*If no data were excluded from the analyses, state so OR if data were excluded, provide the exact number of exclusions and the rationale behind them, indicating whether exclusion criteria were pre-established.*
Non-participation	*State how many participants dropped out/declined participation and the reason(s) given OR provide response rate OR state that no participants dropped out/declined participation.*
Randomization	*If participants were not allocated into experimental groups, state so OR describe how participants were allocated to groups, and if allocation was not random, describe how covariates were controlled.*

Ecological, evolutionary & environmental sciences study design

All studies must disclose on these points even when the disclosure is negative.

Study description	*Briefly describe the study. For quantitative data include treatment factors and interactions, design structure (e.g. factorial, nested, hierarchical), nature and number of experimental units and replicates.*
Research sample	*Describe the research sample (e.g. a group of tagged Passer domesticus, all Stenocereus thurberi within Organ Pipe Cactus National Monument), and provide a rationale for the sample choice. When relevant, describe the organism taxa, source, sex, age range and any manipulations. State what population the sample is meant to represent when applicable. For studies involving existing datasets, describe the data and its source.*
Sampling strategy	*Note the sampling procedure. Describe the statistical methods that were used to predetermine sample size OR if no sample-size calculation was performed, describe how sample sizes were chosen and provide a rationale for why these sample sizes are sufficient.*
Data collection	*Describe the data collection procedure, including who recorded the data and how.*
Timing and spatial scale	*Indicate the start and stop dates of data collection, noting the frequency and periodicity of sampling and providing a rationale for these choices. If there is a gap between collection periods, state the dates for each sample cohort. Specify the spatial scale from which the data are taken*
Data exclusions	*If no data were excluded from the analyses, state so OR if data were excluded, describe the exclusions and the rationale behind them, indicating whether exclusion criteria were pre-established.*
Reproducibility	*Describe the measures taken to verify the reproducibility of experimental findings. For each experiment, note whether any attempts to repeat the experiment failed OR state that all attempts to repeat the experiment were successful.*
Randomization	*Describe how samples/organisms/participants were allocated into groups. If allocation was not random, describe how covariates were controlled. If this is not relevant to your study, explain why.*

Blinding | *Describe the extent of blinding used during data acquisition and analysis. If blinding was not possible, describe why OR explain why blinding was not relevant to your study.*

Did the study involve field work? ☐ Yes ☐ No

Field work, collection and transport

Field conditions	*Describe the study conditions for field work, providing relevant parameters (e.g. temperature, rainfall).*
Location	*State the location of the sampling or experiment, providing relevant parameters (e.g. latitude and longitude, elevation, water depth).*
Access & import/export	*Describe the efforts you have made to access habitats and to collect and import/export your samples in a responsible manner and in compliance with local, national and international laws, noting any permits that were obtained (give the name of the issuing authority, the date of issue, and any identifying information).*
Disturbance	*Describe any disturbance caused by the study and how it was minimized.*

Reporting for specific materials, systems and methods

We require information from authors about some types of materials, experimental systems and methods used in many studies. Here, indicate whether each material, system or method listed is relevant to your study. If you are not sure if a list item applies to your research, read the appropriate section before selecting a response.

Materials & experimental systems

n/a	Involved in the study	
☐	☒	Antibodies
☐	☒	Eukaryotic cell lines
☒	☐	Palaeontology and archaeology
☐	☒	Animals and other organisms
☒	☐	Clinical data
☒	☐	Dual use research of concern
☒	☐	Plants

Methods

n/a	Involved in the study	
☐	☒	ChIP-seq
☒	☐	Flow cytometry
☒	☐	MRI-based neuroimaging

Antibodies

Antibodies used

WB and IF: ZEB1 (1:2000 WB, 1:250 IF, Sigma, HPA027524), Vimentin (1:2000 WB, 1:250 IF, Cell Signaling; #5741), E-Cadherin (1:5000 WB, 1:250 IF, BD Biosciences, 610182)
WB: β-Actin (1:10000, Sigma, A5441), ACLS4 (1:1000, Santa Cruz, sc-271800), GAPDH (1:10000, Cell Signaling, 2118), SNAIL (1:500, Cell signaling, 3895), TWIST (1:1000, abcam, ab50581), ELOVL5 (1:1000, PA583879, Thermo)
WB and IHC: SCD (1:1000 WB, 1:250 IHC, Proteintech, 23393-1-AP), FADS2 (1:1000 WB,1:100 IHC, Proteintech, 28034-1-AP), FASN (1:1000 WB,1:50 IHC, MA5-14887, Thermo)
IHC: ELOVL5 (1:100, Novus, NBP3-14304), ZEB1 (1:150, Novus, NBP1-05987), ACSL4 (1:250, Santa Cruz, sc365230), 4-HNE (1:250, Abcam ab46545)
Secondary antibodies:
for WB: goat anti-Mouse IgG Peroxidase (1:10000, #115-035-1463, Jackson ImmunoResearch); goat anti-rabbit IgG Peroxidase (1:10000, #111-035-144, Jackson ImmunoResearch)
for IF: AlexaFluor 555 goat anti-rabbit IgG (H+L) (1:300, #A21428, Life Technologies); AlexaFluor 488 goat anti-mouse IgG (H+L) (1:300, #A11029, Life Technologies)

Validation

Antibody validation information can be found on manufacturer's websites.

Eukaryotic cell lines

Policy information about [cell lines and Sex and Gender in Research](#)

Cell line source(s)	All human established cancer cell lines (MDA-MB-231, MCF7, H358, A549, BxPC3, 143B and U2OS) were purchased from the American Type Culture Collection (ATCC). Generation of murine KPC cell lines was described in Krebs et al. Nature Cell Biology (2017).
Authentication	cells have not been authenticated.

Mycoplasma contamination	cells were routinely tested for mycoplasma contamination by MycoAlert Mycoplasma Detection Kit (LT07-218; Lonza) and no contamination was observed throughout the stud
Commonly misidentified lines (See ICLAC register)	No commonly misidentified lines were used.

Palaeontology and Archaeology

Specimen provenance	*Provide provenance information for specimens and describe permits that were obtained for the work (including the name of the issuing authority, the date of issue, and any identifying information). Permits should encompass collection and, where applicable, export.*
Specimen deposition	*Indicate where the specimens have been deposited to permit free access by other researchers.*
Dating methods	*If new dates are provided, describe how they were obtained (e.g. collection, storage, sample pretreatment and measurement), where they were obtained (i.e. lab name), the calibration program and the protocol for quality assurance OR state that no new dates are provided.*

☐ Tick this box to confirm that the raw and calibrated dates are available in the paper or in Supplementary Information.

Ethics oversight	*Identify the organization(s) that approved or provided guidance on the study protocol, OR state that no ethical approval or guidance was required and explain why not.*

Note that full information on the approval of the study protocol must also be provided in the manuscript.

Animals and other research organisms

Policy information about studies involving animals; ARRIVE guidelines recommended for reporting animal research, and Sex and Gender in Research

Laboratory animals	All mice were 8-week old C57BL/6NRj mice and purchased from Janvier labs. For lung colonization experiments, littermates of both sexes were used, for PCLS experiments only female mice were used.<br>Mice were kept in individually ventilated cages according to European welfare regulations 2010/63, providing constant temperature between 20-24°C and 45-65% humidity and kept on a 12:12 h light-dark cycle.
Wild animals	The study did not involve wild animals.
Reporting on sex	This study did not focus on sex differences. Cohorts for experiments were chosen by availability and age, independent of the sex. Nevertheless, roughly same numbers of males and females were used for each experiment.
Field-collected samples	No field-collected samples were used in this study.
Ethics oversight	Animal husbandry and all experiments were performed according to the European Animal Welfare laws and guidelines. The protocols were approved by the committee of ethics of animal experiments of the state of Bavaria (Regierung Unterfranken, Würzburg; Regierung von Unterfranken, Würzburg; TS-30-2021, 55.2DMS2532-2-1832)

Note that full information on the approval of the study protocol must also be provided in the manuscript.

Clinical data

Policy information about clinical studies

All manuscripts should comply with the ICMJE guidelines for publication of clinical research and a completed CONSORT checklist must be included with all submissions.

Clinical trial registration	*Provide the trial registration number from ClinicalTrials.gov or an equivalent agency.*
Study protocol	*Note where the full trial protocol can be accessed OR if not available, explain why.*
Data collection	*Describe the settings and locales of data collection, noting the time periods of recruitment and data collection.*
Outcomes	*Describe how you pre-defined primary and secondary outcome measures and how you assessed these measures.*

Dual use research of concern

Policy information about dual use research of concern

Hazards

Could the accidental, deliberate or reckless misuse of agents or technologies generated in the work, or the application of information presented in the manuscript, pose a threat to:

No	Yes	
☐	☐	Public health
☐	☐	National security
☐	☐	Crops and/or livestock
☐	☐	Ecosystems
☐	☐	Any other significant area

Experiments of concern

Does the work involve any of these experiments of concern:

No	Yes	
☐	☐	Demonstrate how to render a vaccine ineffective
☐	☐	Confer resistance to therapeutically useful antibiotics or antiviral agents
☐	☐	Enhance the virulence of a pathogen or render a nonpathogen virulent
☐	☐	Increase transmissibility of a pathogen
☐	☐	Alter the host range of a pathogen
☐	☐	Enable evasion of diagnostic/detection modalities
☐	☐	Enable the weaponization of a biological agent or toxin
☐	☐	Any other potentially harmful combination of experiments and agents

Plants

Seed stocks	n/a
Novel plant genotypes	n/a
Authentication	n/a

ChIP-seq

Data deposition

☒ Confirm that both raw and final processed data have been deposited in a public database such as GEO.

☐ Confirm that you have deposited or provided access to graph files (e.g. BED files) for the called peaks.

Data access links<br>*May remain private before publication.*	GSE264671<br>https://www.ncbi.nlm.nih.gov/geo/
Files in database submission	MDA_shGFP1_H3K27Ac_R1.fastq.gz, MDA_shGFP1_H3K27Ac_R2.fastq.gz, MDA_shGFP4_H3K27Ac_R1.fastq.gz, MDA_shGFP4_H3K27Ac_R2.fastq.gz, MDA_shZEB13_H3K27Ac_R1.fastq.gz, MDA_shZEB13_H3K27Ac_R2.fastq.gz, MDA_shZEBF10_H3K27Ac_R1.fastq.gz, MDA_shZEBF10_H3K27Ac_R2.fastq.gz
Genome browser session<br>(e.g. UCSC)	N/A

Methodology

Replicates	Two biological replicates for each condition, representing control and ZEB1 knockdown conditions.
Sequencing depth	20 milion reads.
Antibodies	rabbit anti-trimethyl histone H3K27Ac, Millipore 07-449
Peak calling parameters	No peaks were called.
Data quality	Raw reads quality by FASTQC sofware, and low-quality reads were removed and the reads contaminated with adaptor sequences were trimmed.
Software	Raw reads were quality filtered and adapter-trimmed using cutadapt v.1.9.1, then aligned to GRCh38 ensemble genome assembly.

Software | using Bowtie2 v. 2.2.9. BigWig files were generated using deeptools v 2.3.

Flow Cytometry

Plots

Confirm that:

- [ ] The axis labels state the marker and fluorochrome used (e.g. CD4-FITC).
- [ ] The axis scales are clearly visible. Include numbers along axes only for bottom left plot of group (a 'group' is an analysis of identical markers).
- [ ] All plots are contour plots with outliers or pseudocolor plots.
- [ ] A numerical value for number of cells or percentage (with statistics) is provided.

Methodology

Sample preparation: *Describe the sample preparation, detailing the biological source of the cells and any tissue processing steps used.*

Instrument: *Identify the instrument used for data collection, specifying make and model number.*

Software: *Describe the software used to collect and analyze the flow cytometry data. For custom code that has been deposited into a community repository, provide accession details.*

Cell population abundance: *Describe the abundance of the relevant cell populations within post-sort fractions, providing details on the purity of the samples and how it was determined.*

Gating strategy: *Describe the gating strategy used for all relevant experiments, specifying the preliminary FSC/SSC gates of the starting cell population, indicating where boundaries between "positive" and "negative" staining cell populations are defined.*

- [ ] Tick this box to confirm that a figure exemplifying the gating strategy is provided in the Supplementary Information.

Magnetic resonance imaging

Experimental design

Design type: *Indicate task or resting state; event-related or block design.*

Design specifications: *Specify the number of blocks, trials or experimental units per session and/or subject, and specify the length of each trial or block (if trials are blocked) and interval between trials.*

Behavioral performance measures: *State number and/or type of variables recorded (e.g. correct button press, response time) and what statistics were used to establish that the subjects were performing the task as expected (e.g. mean, range, and/or standard deviation across subjects).*

Acquisition

Imaging type(s): *Specify: functional, structural, diffusion, perfusion.*

Field strength: *Specify in Tesla*

Sequence & imaging parameters: *Specify the pulse sequence type (gradient echo, spin echo, etc.), imaging type (EPI, spiral, etc.), field of view, matrix size, slice thickness, orientation and TE/TR/flip angle.*

Area of acquisition: *State whether a whole brain scan was used OR define the area of acquisition, describing how the region was determined.*

Diffusion MRI: [ ] Used [ ] Not used

Preprocessing

Preprocessing software: *Provide detail on software version and revision number and on specific parameters (model/functions, brain extraction, segmentation, smoothing kernel size, etc.).*

Normalization: *If data were normalized/standardized, describe the approach(es): specify linear or non-linear and define image types used for transformation OR indicate that data were not normalized and explain rationale for lack of normalization.*

Normalization template: *Describe the template used for normalization/transformation, specifying subject space or group standardized space (e.g. original Talairach, MNI305, ICBM152) OR indicate that the data were not normalized.*

Noise and artifact removal: *Describe your procedure(s) for artifact and structured noise removal, specifying motion parameters, tissue signals and*

Noise and artifact removal | physiological signals (heart rate, respiration).

Volume censoring | Define your software and/or method and criteria for volume censoring, and state the extent of such censoring.

Statistical modeling & inference

Model type and settings | Specify type (mass univariate, multivariate, RSA, predictive, etc.) and describe essential details of the model at the first and second levels (e.g. fixed, random or mixed effects; drift or auto-correlation).

Effect(s) tested | Define precise effect in terms of the task or stimulus conditions instead of psychological concepts and indicate whether ANOVA or factorial designs were used.

Specify type of analysis: ☐ Whole brain ☐ ROI-based ☐ Both

Statistic type for inference (See Eklund et al. 2016) | Specify voxel-wise or cluster-wise and report all relevant parameters for cluster-wise methods.

Correction | Describe the type of correction and how it is obtained for multiple comparisons (e.g. FWE, FDR, permutation or Monte Carlo).

Models & analysis

n/a	Involved in the study
☐	☐ Functional and/or effective connectivity
☐	☐ Graph analysis
☐	☐ Multivariate modeling or predictive analysis

Functional and/or effective connectivity | Report the measures of dependence used and the model details (e.g. Pearson correlation, partial correlation, mutual information).

Graph analysis | Report the dependent variable and connectivity measure, specifying weighted graph or binarized graph, subject- or group-level, and the global and/or node summaries used (e.g. clustering coefficient, efficiency, etc.).

Multivariate modeling and predictive analysis | Specify independent variables, features extraction and dimension reduction, model, training and evaluation metrics.
