# Supplementary figures and images for: Zeb1 mediates EMT/plasticity-associated ferroptosis sensitivity in cancer cells by regulating lipogenic enzyme expression and phospholipid composition
Source: Nat Cell Biol. 2024 Jul 15;26(9):18. doi: 10.1038/s41556-024-01464-1 (PMC11392809; doi:10.1038/s41556-024-01464-1)

## Slide 1
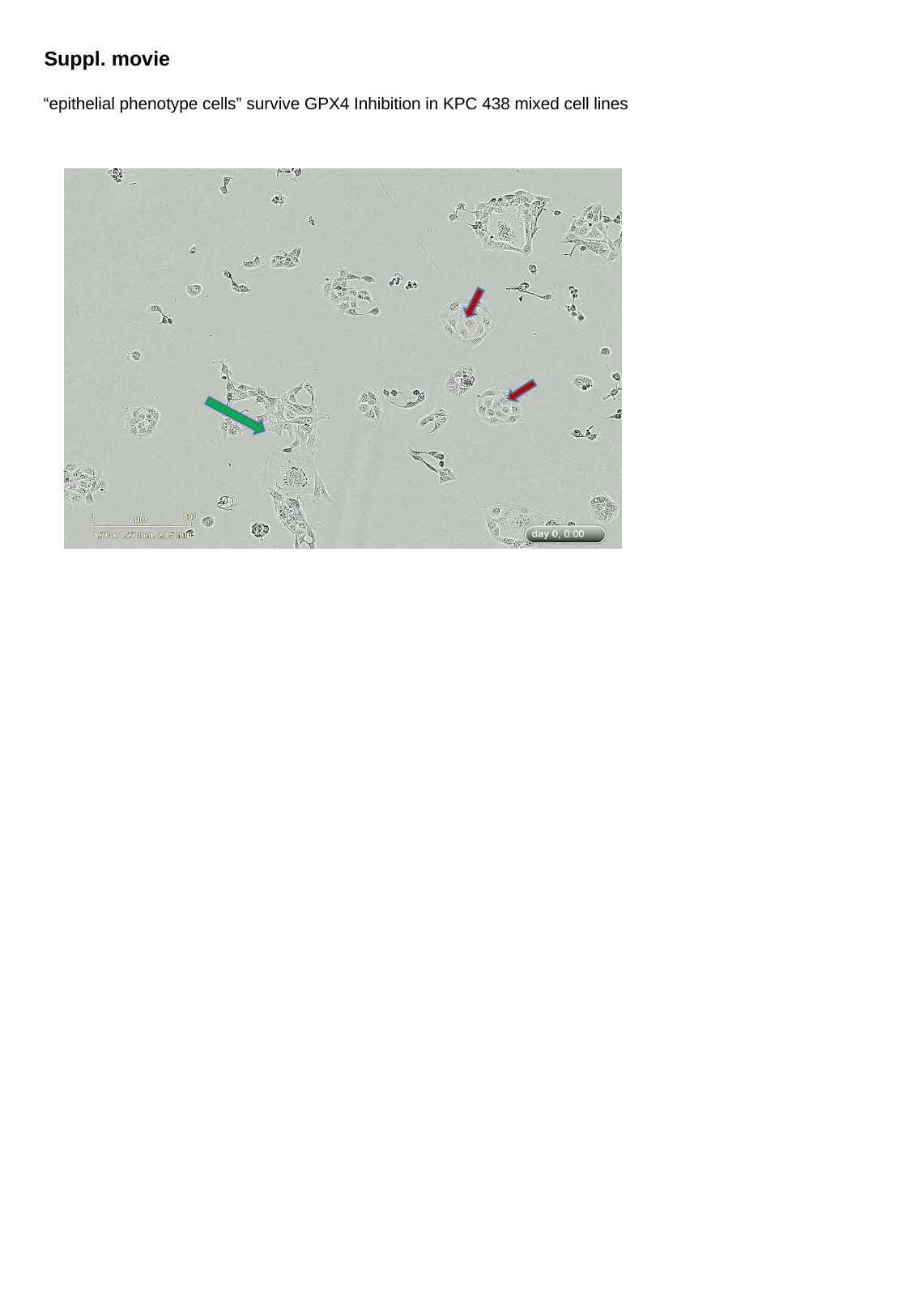

Suppl. movie
“epithelial phenotype cells” survive GPX4 Inhibition in KPC 438 mixed cell lines

Supplement: Supplementary file 2 — Primarily epithelial-type cancer cells survive GPX4 inhibition in mixed cell lines (here KPC438 mixed, treated for 66 h with 16 µM ML210) (red arrows indicate epithelial cancer cells and green arrow indicates mesenchymal cancer cells). [file 41556_2024_1464_MOESM2_ESM.pptx]
