# Supplementary material for: Zeb1 mediates EMT/plasticity-associated ferroptosis sensitivity in cancer cells by regulating lipogenic enzyme expression and phospholipid composition
Source: Nat Cell Biol. 2024 Jul 15;26(9):18. doi: 10.1038/s41556-024-01464-1 (PMC11392809; doi:10.1038/s41556-024-01464-1)

1a)

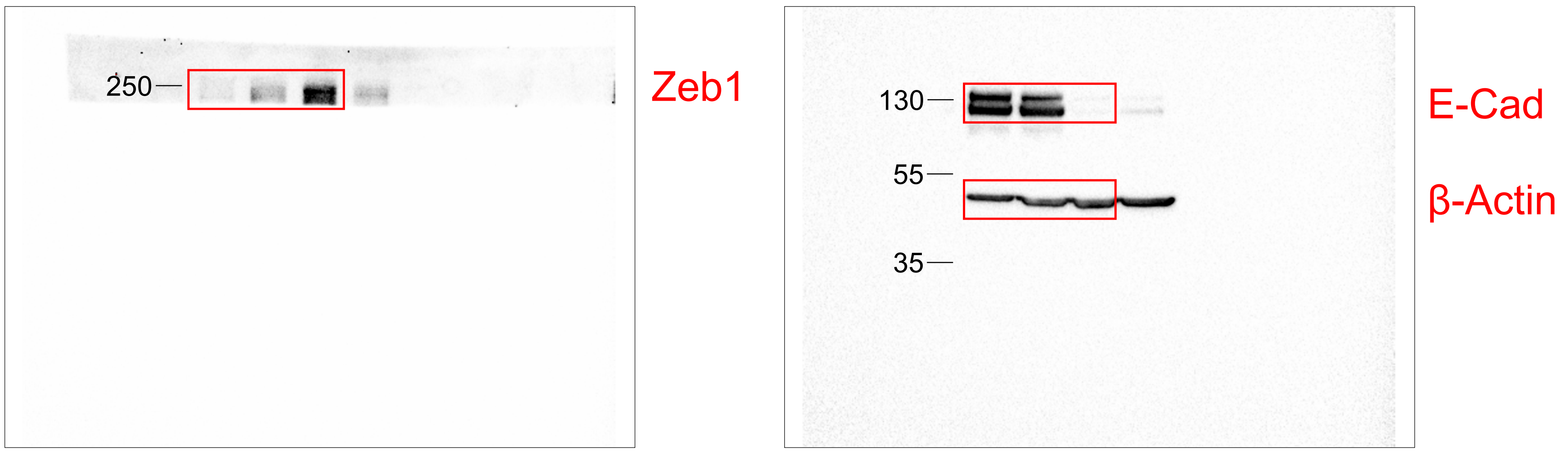
250
Zeb1
130
55
35
E-Cad
β-Actin

1c)

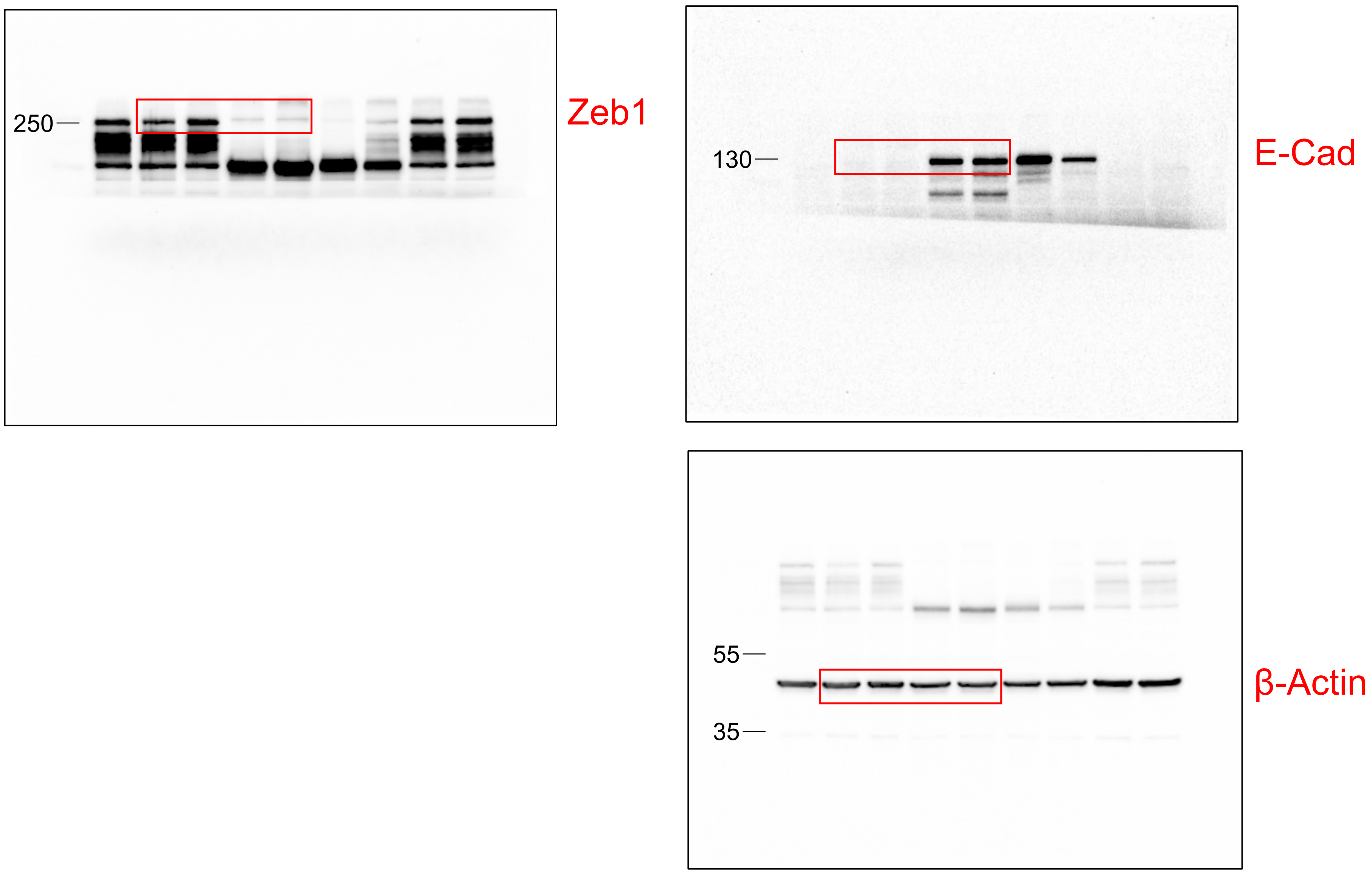
250
Zeb1
130
E-Cad
55
35
β-Actin

1e)

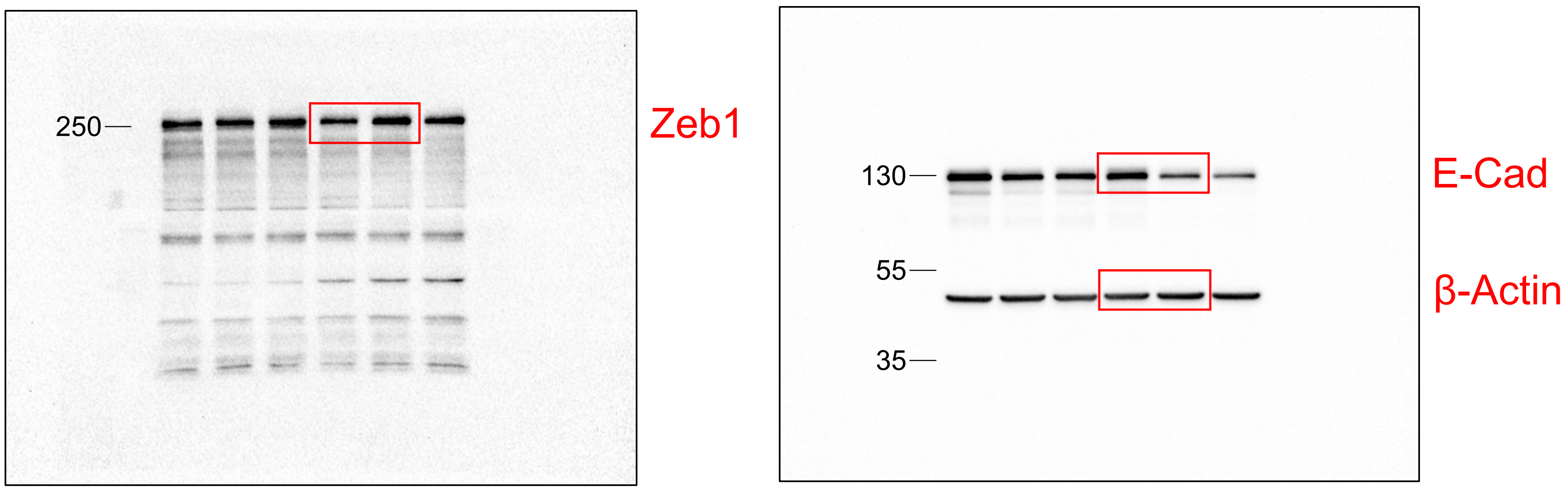
250
Zeb1
130
55
35
E-Cad
β-Actin

1f)

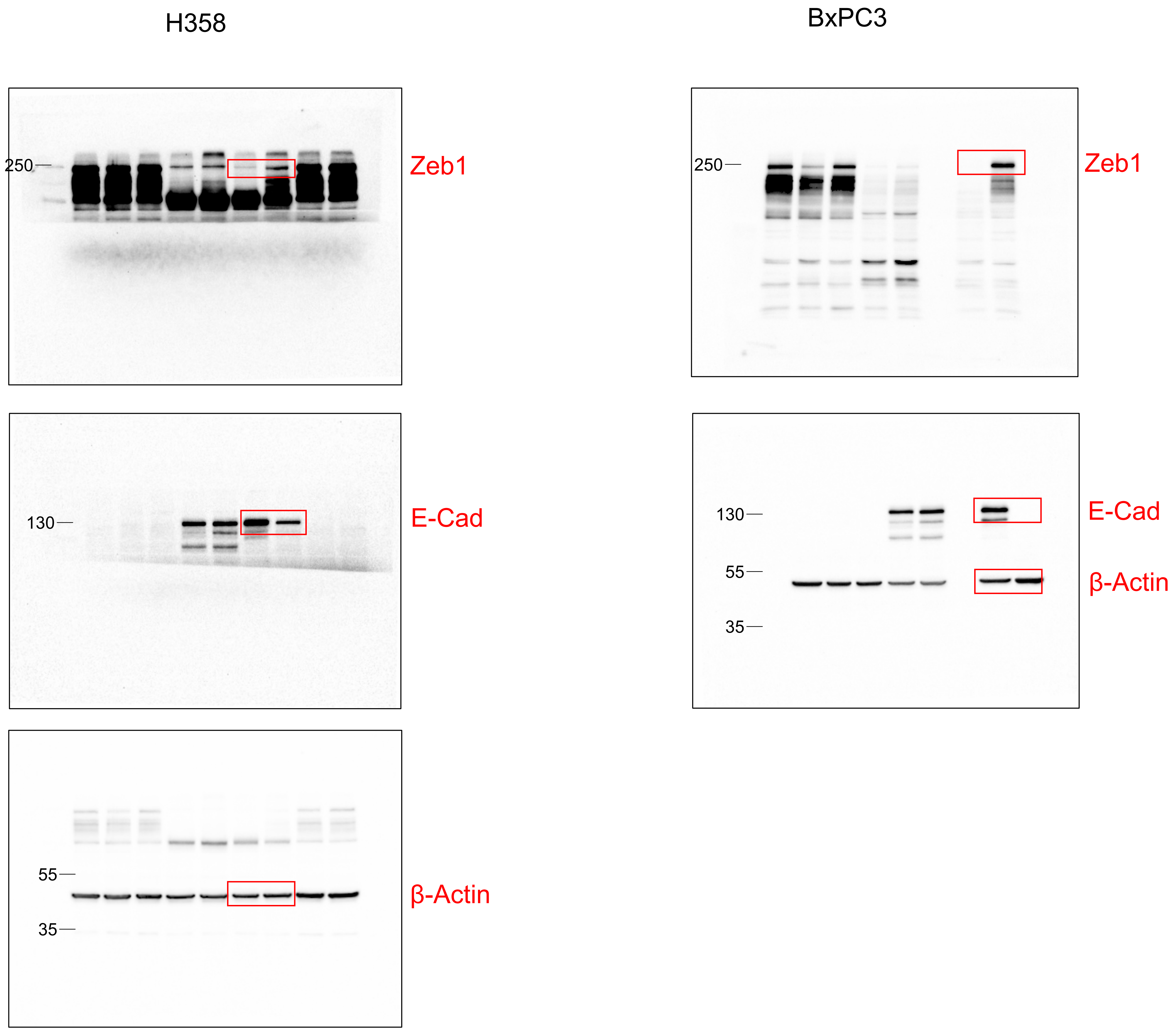
H358
BxPC3
250
Zeb1
130
E-Cad
55
35
β-Actin
250
Zeb1
130
55
35
E-Cad
β-Actin

3b)

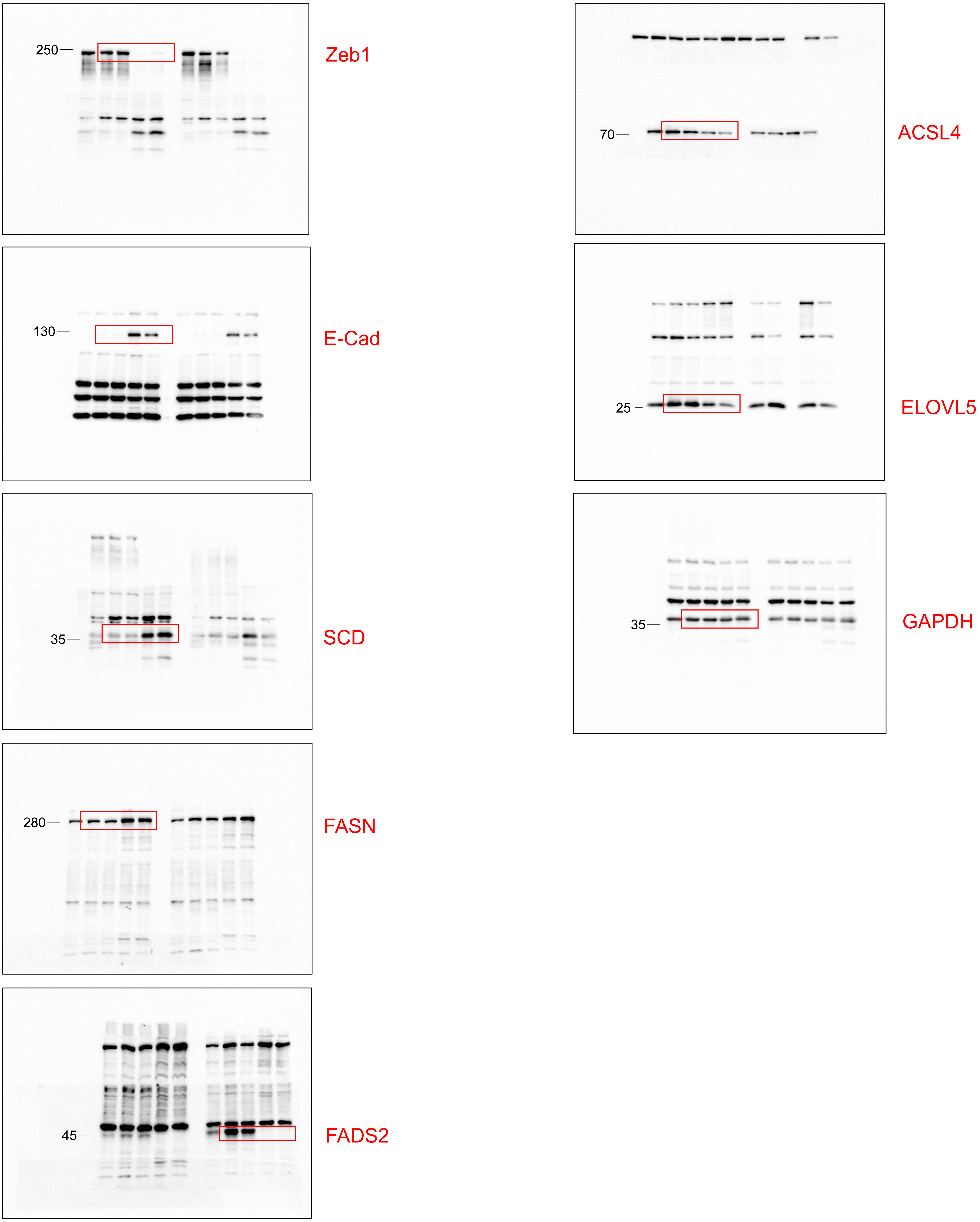
250
Zeb1
130
E-Cad
35
SCD
280
FASN
45
FADS2
70
ACSL4
25
ELOVL5
35
GAPDH

Extended Fig. 1a)

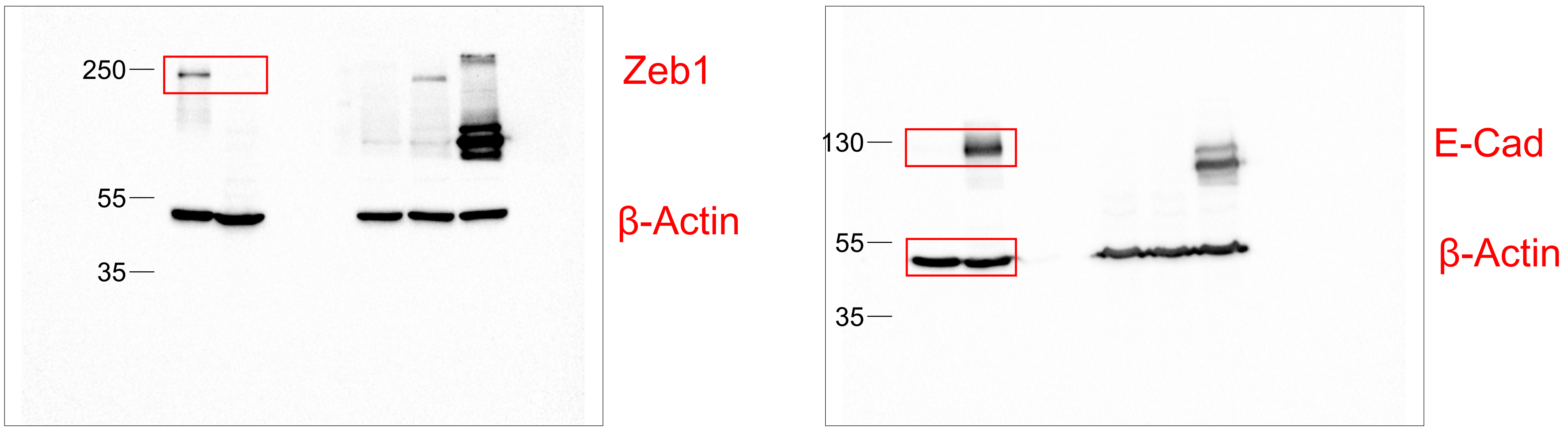

Extended Fig. 1b)

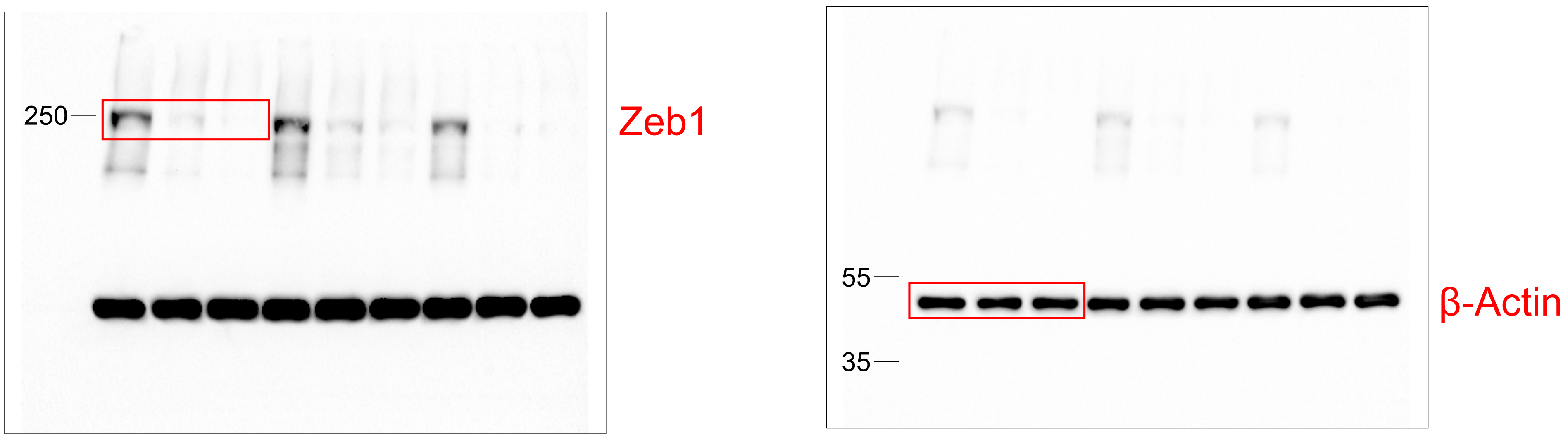

Extended Fig. 1c)

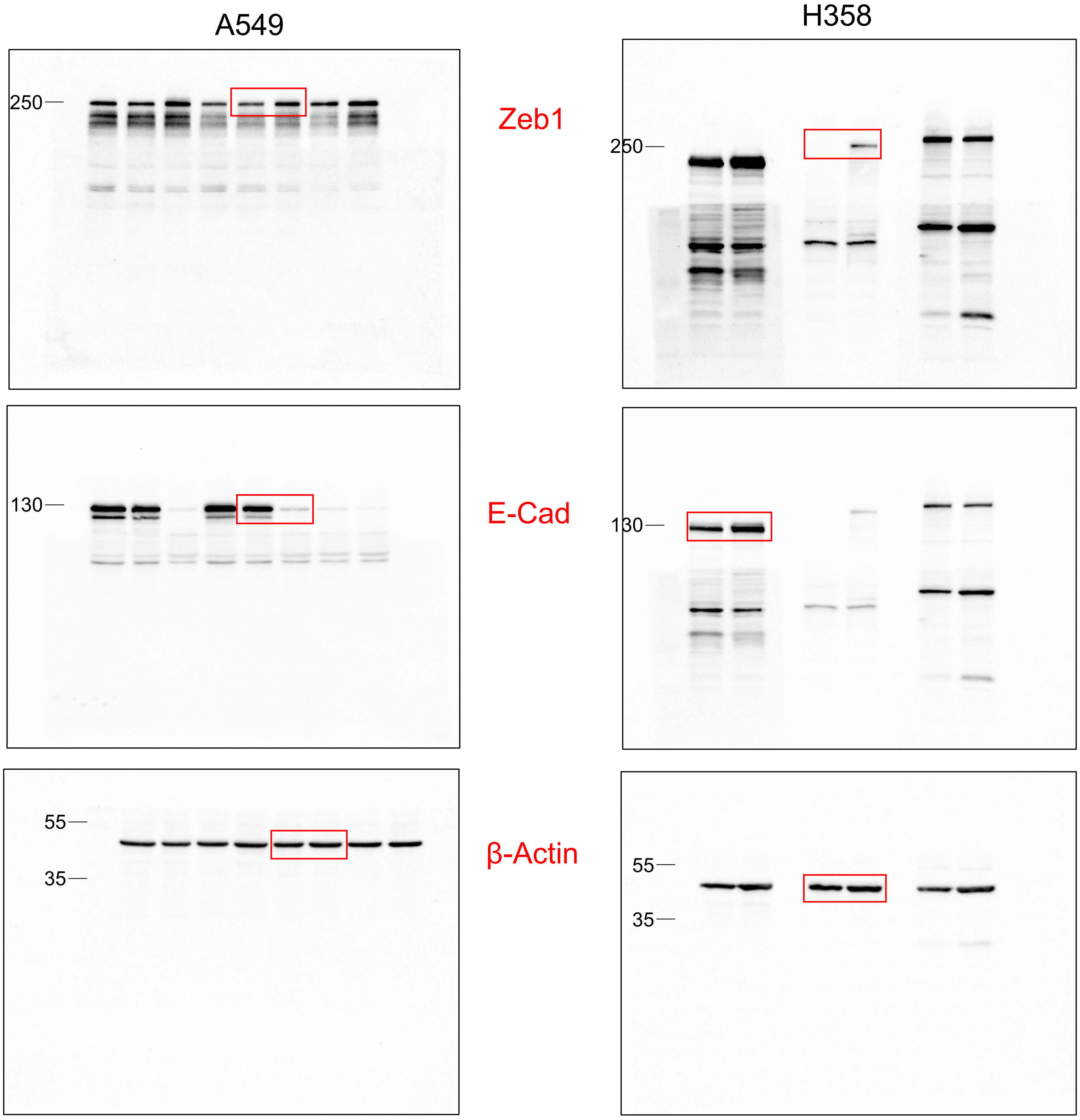

Extended Fig. 1d)

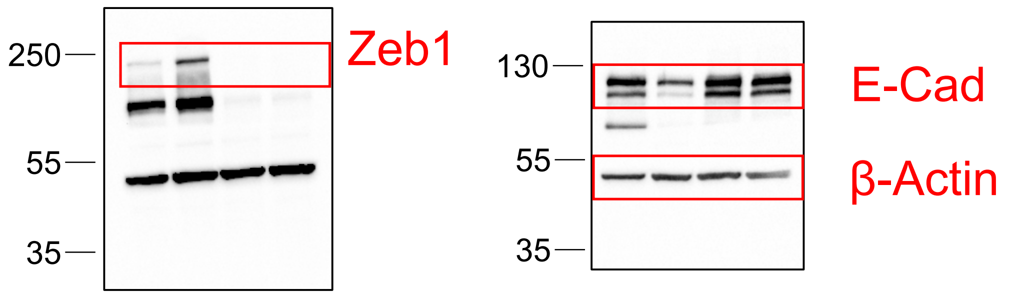

Extended Fig. 1f)

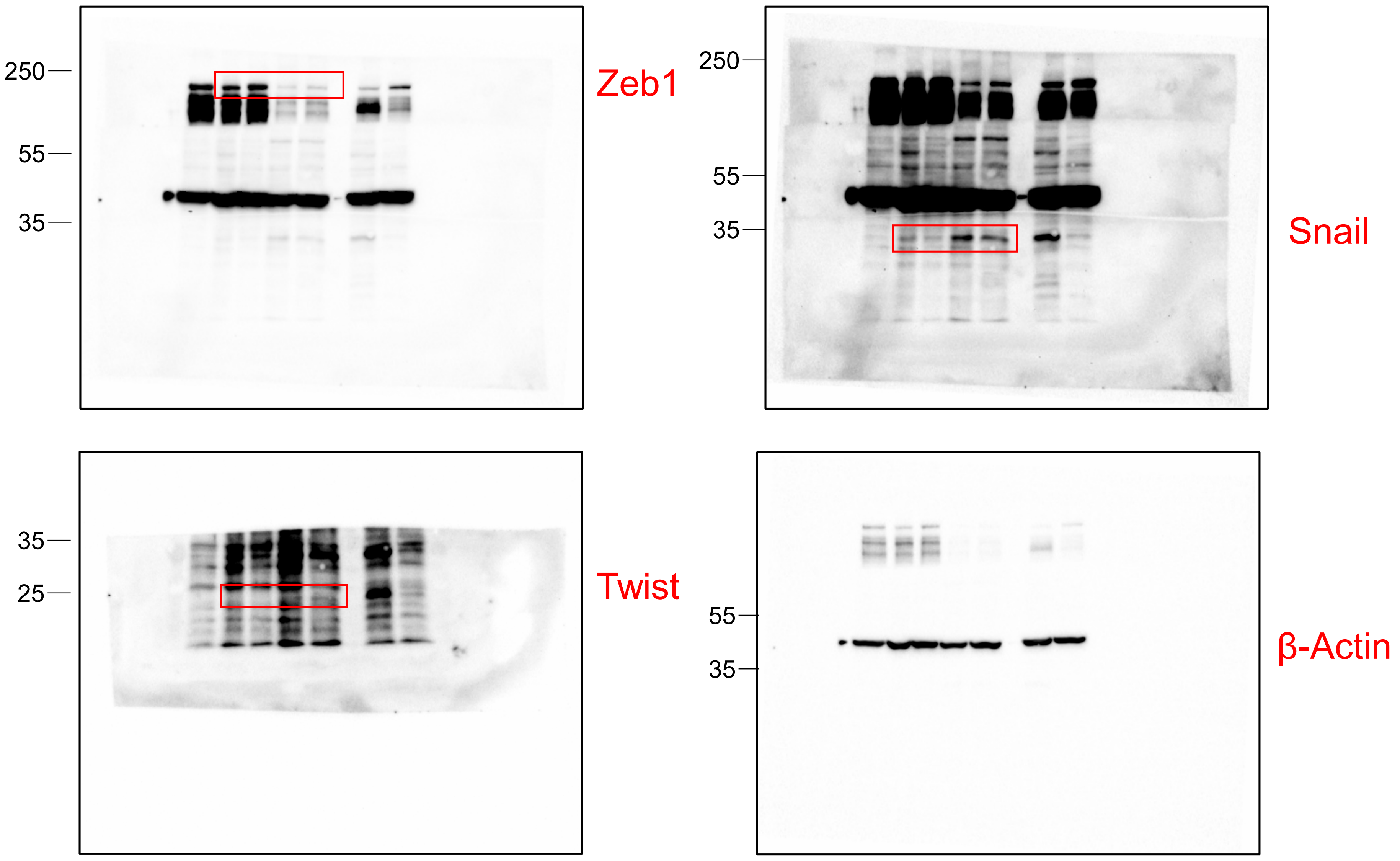

Extended Fig. 6f)

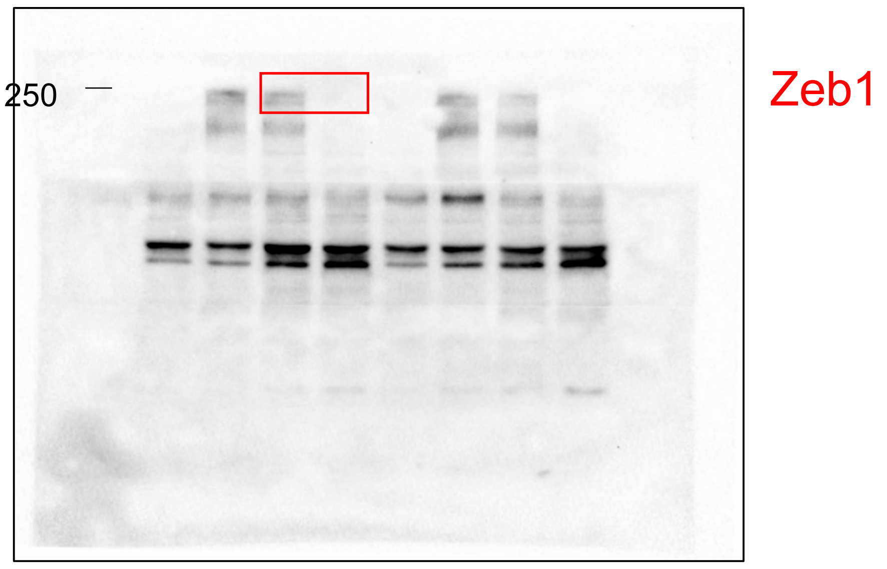

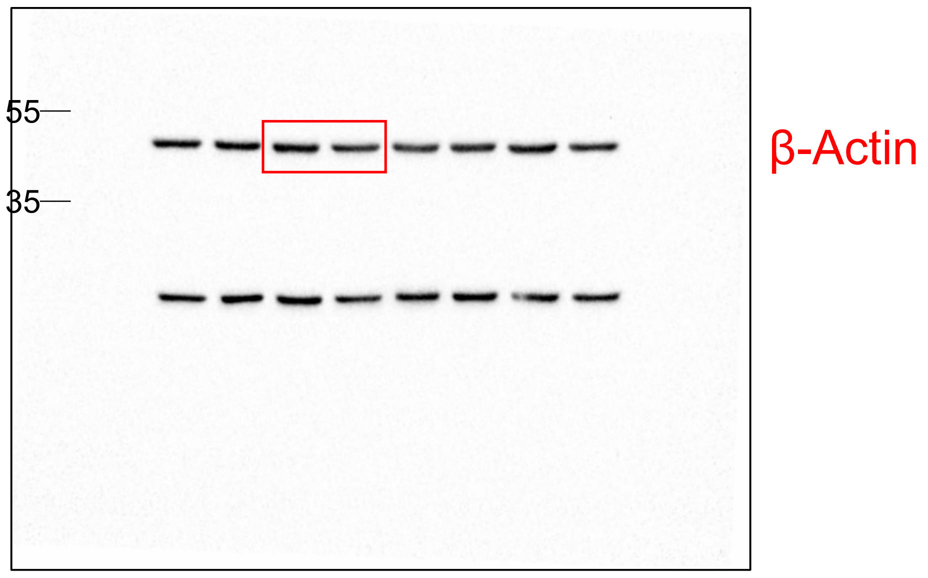

Extended Fig. 6g)

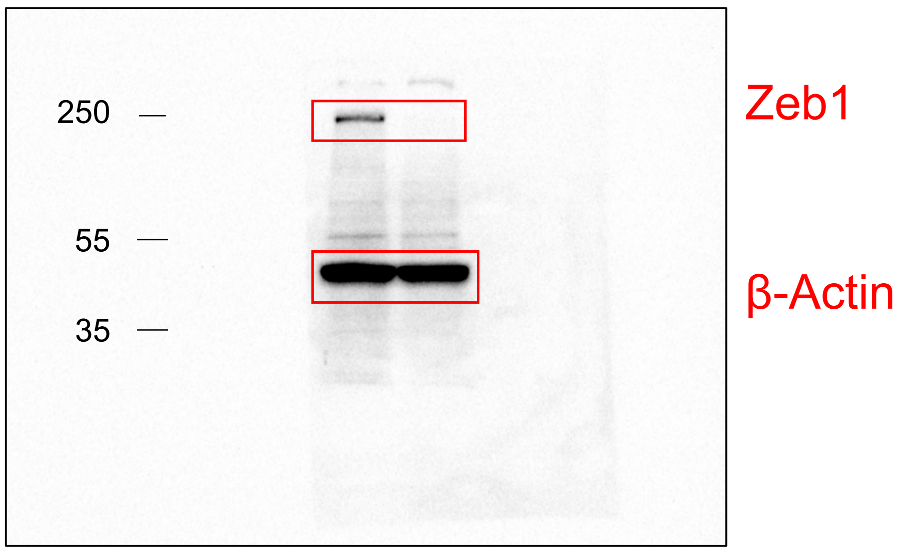

Supplement: Supplementary file 5 — All unprocessed blots with clearly labelled blots for each item. [file 41556_2024_1464_MOESM5_ESM.pdf]
